# Impact of student-induced disturbance on stream macroinvertebrates differs among habitat types

**DOI:** 10.1038/s41598-018-38210-1

**Published:** 2019-02-05

**Authors:** Jon P. Bossley, Peter C. Smiley

**Affiliations:** 1 Environmental Science Graduate Program, The Ohio State University, 174 West 18th, Columbus, OH 43210 USA; 2 School of Natural and Social Sciences, Mount Vernon Nazarene University, 800 Martinsburg Road, Mount Vernon, OH 43050 USA; 3 USDA Agricultural Research Service, Soil Drainage Research Unit, 590 Woody Hayes Drive, Columbus, OH 43210 USA

## Abstract

Environmental impacts from ecotourism and outdoor recreation activities on terrestrial and aquatic ecosystems are well-reported in the literature, but less is known regarding the impacts of outdoor environmental education activities. Student activity during stream classes may cause substrate disruption and localized impacts on stream macroinvertebrates. We hypothesized that student activity would negatively impact macroinvertebrate community structure in three habitat types (riffle, run, pool) within a site regularly used for stream classes while no impact from student activity would occur in the same three habitat types within an unused site. We addressed the hypothesis by sampling macroinvertebrates monthly for one year in the riffles, runs, and pools at the class site and the unused site within a fourth order stream in central Ohio. The results indicated reduced macroinvertebrate abundance and richness in the riffle at the class site during periods with student activity and no differences between site types during periods without student activity. No impacts of stream classes on macroinvertebrate communities were observed in runs or pools. The results suggest that environmental education organizations should avoid repetitive use of the same site for their stream classes to avoid reductions of macroinvertebrate abundance and taxa richness that can impact the students’ educational experience.

## Introduction

The rising popularity of ecotourism has resulted in millions of tourists visiting natural areas across the globe annually^1–3^. Despite the benefits of ecotourism to host communities, tourists, and the local flora and fauna, researchers and industry professionals have observed environmental problems stemming from mass tourism^2^. Negative impacts of ecotourism at high-use sites have been documented globally across a wide range of terrestrial and aquatic ecosystems^4^. Notable examples in marine ecosystems include negative impacts on: (1) coral reefs in Brazil and the Philippines due to diving activities^5–7^; (2) shorebirds in Australia as a result of beachside driving^8^; and (3) bottlenose dolphin populations in New Zealand due to dolphin watching excursions^9^.

Similarly, the impacts of recreational activities have been documented in numerous freshwater ecosystems^4,10^. Water-based recreational activities have been shown to cause physical habitat degradation, chemical pollution, and impacts to aquatic plants, vertebrates, and macroinvertebrates^10,11^. Specifically, macroinvertebrates are of interest to scientists because they are effective bioindicators of anthropogenic impacts and they play an integral role in the aquatic food web as detrivores and prey^12^. Experimental disturbances, instream recreational activities, and recreational stream crossings have been documented to negatively impact the population and community structure of freshwater macroinvertebrates. Experiments in Australia and Brazil that simulated instream trampling by hikers and bathers revealed changes in macroinvertebrate taxa composition and reductions in taxa richness and abundance^13,14^. Caires *et al*.^15^ observed an increase in drifting behavior in *Baetis* mayflies in a Utah river in response to instream hiking, but found no effect of instream hiking on macroinvertebrate abundance, taxa richness, and the abundance of the most common taxa. Wright & Li^16^ observed reductions in densities of caddisfly (*Dicosmoecus gilvipes*) larvae in Oregon due to recreational activities including streamside camping, fishing, goldpanning, swimming, and tubing. Laing^17^ noted decreased invertebrate abundance, insect abundance, Ephemeroptera Plecoptera Trichoptera (EPT) abundance, and taxa richness within tributaries of a scenic river in Nebraska due to canoers hiking through the water.

Impacts on aquatic macroinvertebrates from recreational stream crossings have also been documented^18–20^. Kidd *et al*.^18^ observed decreased water quality, taxa richness, percent EPT (excluding Hydropsychidae), and percent clingers and increased percentages of Chironomidae and Oligochaeta below stream crossings in Virginia. Conversely, Heth *et al*.^19^ found that taxa richness, EPT richness, Shannon diversity, and biotic index scores increased downstream of crossings in a Missouri stream in the summer and that no differences occurred in the winter. Holmquist *et al*.^20^ also observed increased invertebrate abundance, species richness, dominance, percent tolerant fauna, Chironomidae abundance, and Hilsenhoff biotic index scores below stream crossings in California streams in the late summer.

While effects of ecotourism and outdoor recreation on the environment have been well-studied, environmental education programs can impose similar impacts but have been far less studied. Ecotourism and outdoor recreation encompass activities in which participants engage by choice during their leisure time^2,4,10^. In contrast, environmental education activities are generally more structured and conducted as a regular component of the school curriculum via in-class lab activities, day-long field trips to nature centers and parks, and multi-day stays at resident outdoor education (ROE) centers. Such parks, nature centers, and ROE centers may host thousands of students over the course of an academic year^21,22^. Classes that focus on stream biomonitoring and/or involve stream exploration have become a popular component in many of these educational programs^23^. Aquatic activities and classes are typically conducted in the same body of water and often at the same site due to ease of access, safety, and/or tradition^22^. Consequently, organizations that serve large clienteles may unknowingly and inadvertently cause environmental disturbance in the same aquatic environment their activities and classes are intended to promote. However, empirical data to support this concern are presently limited.

As part of a series of four studies, Bossley & Smiley^21,22,24^ investigated the effects of ROE stream classes on aquatic macroinvertebrates in agricultural streams in central Ohio. Bossley & Smiley^21^ found that within a fourth order stream student instream activity did not alter short-term temporal trends in macroinvertebrate community structure in a riffle regularly used for ROE stream classes. Additionally, differences in macroinvertebrate community structure between the class riffle and a riffle unused by ROE stream classes were observed^21^. Bossley & Smiley^22^ also observed substrate rearrangement by students in ROE stream classes and found greater rock movement in the class riffle compared to an upstream riffle unaffected by student activity. Also, abundance, richness, EPT abundance, and clinger abundance were greater on rocks in the unused riffle than in the class riffle at the end of the six-week study^22^. These initial findings^21,22^ documenting spatial differences in macroinvertebrate community structure suggest the potential long-term impacts of student instream activity within this fourth order stream. In contrast, an investigation of macroinvertebrate community response to simulated substrate disturbance in riffles within headwater tributaries unused by ROE stream classes in central Ohio revealed no effect of trampling on macroinvertebrates^24^.

Within this manuscript we present the results of the fourth study that consists of a year-long investigation of trends in macroinvertebrate community structure across three habitat types (riffle, run, pool) and four seasons between the class site and the unused site. Previous studies^13–22,24^ evaluating the impact of recreational activities and ROE classes in streams were mostly short term studies that encompassed one to three seasons. Additionally, previous studies evaluating the impacts of recreational activities and ROE classes in streams focused exclusively on riffles^13,15,19–22,24^ or conducted reach level assessments involving composited information from multiple habitat types^14,16–18^ and did not assess whether the anthropogenic impact on macroinvertebrates differed among habitat types. Habitat types in streams (i.e., riffles, runs, pools) differ in water depth, velocity, and substrate types^25–27^. It has also been well documented that macroinvertebrate abundance, diversity, and taxa composition also differ among habitat types within streams in North America, South America, Europe, Japan, Australia, and New Zealand^28–33^. Macroinvertebrates exhibit distinct preferences for substrate size and stability, and habitat types having greater substrate heterogeneity often exhibit greater macroinvertebrate biodiversity^34^. These documented differences in habitat characteristics and macroinvertebrate community structure among habitat types in streams suggest that the effects of disturbance on macroinvertebrates may differ among habitat types^35,36^.

Previous studies^30,33,37–39^ documented that macroinvertebrate responses to flooding and land use change differ among stream habitat types. Macroinvertebrate density, taxa richness, and taxa composition exhibited habitat specific responses to a spate in a second order stream in Japan^30^. Macroinvertebrate density decreased during the spate and then increased after the spate in riffles, but did not change during or after the spate in pools, glides, and backwaters^30^. Taxa richness decreased in pools after the spate, but did not change in riffles, glides, or backwaters^30^. Taxa composition changed in riffles and backwaters after the spate, but not in glides and pools^30^. Decreases in macroinvertebrate abundance after floods were the least in physically stable bedrock habitats and greatest in pools and runs in a river in Switzerland^37^. Percent EPT and percent Baetidae, Hydropyschidae, and Heptageniidae decreased in riffles after a spate, while percent EPT and percent Chironomidae increased in pools after a spate in a third order stream in Ohio^38^. Abundance and diversity of macroinvertebrates in riffles exhibited stronger relationships with physical and chemical variables associated with land use change than the abundance and diversity in pools and bank habitats in sand gravel streams in Georgia^39^. EPT taxa richness and Shannon diversity index in riffles were more sensitive indicators of land use change in riffles than in pools in headwater streams in Brazil^33^. However, whether the impacts of recreation and outdoor education activities on macroinvertebrates differ among riffles, runs, and pools is unknown.

Prior observations of stream classes at Heartland Outdoor School (Heartland) documented that student instream activity involves physical disruption of the stream substrate throughout a stretch of stream encompassing riffle, run, and pool habitats^23^. Based on these observations, we hypothesized that ROE stream classes will negatively impact macroinvertebrate community structure within the riffle, run, and pool habitat types within the stream site regularly used by ROE stream classes during time periods when stream classes are in session. However, no impact will occur during time periods when stream classes are not in session. We addressed the hypothesis by conducting a year-long study that involved monthly sampling of aquatic macroinvertebrates in riffles, runs, and pools within the class site and an unused site within a fourth order stream. Additionally, to address the hypothesis we ensured that sampling was conducted during periods with student activity and periods without student activity.

## Results

### Riffles

**Figure 1:**
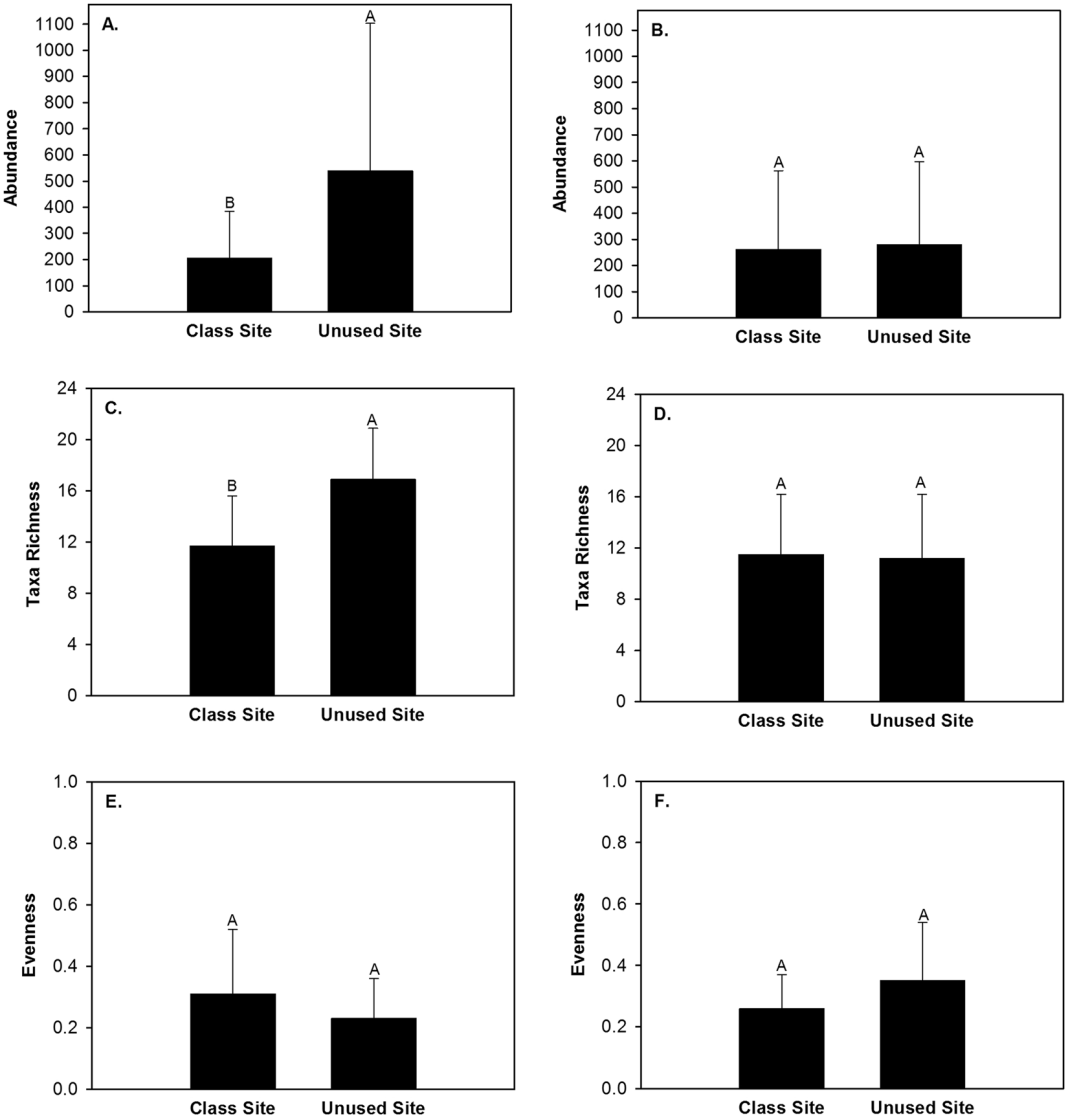
Mean and standard deviation of macroinvertebrate abundance (**A,B**), taxa richness (**C,D**), and evenness (**E,F**) within riffles in the class site and the unused site during time periods with student activity (**A,C,E**) and without student activity (**B,D,F**), Alum Creek, Ohio, February 2013 to January 2014.

During the 12 month study, 21,911 macroinvertebrates and 56 taxa were captured within riffles (Table 1). The five most abundant taxa constituted 80.6% of all captures in riffles and consisted of Chironomidae, Hydropsychidae, Turbellaria, Elmidae, and Simuliidae (Table 1). Macroinvertebrate abundance, taxa richness, and evenness in riffles exhibited a significant interaction effect of site type and student activity period (Table 2). During time periods with student activity macroinvertebrate abundance and taxa richness were greater in the riffle in the unused site than the class site (Fig. 1). During time periods without student activity macroinvertebrate abundance and taxa richness in the riffles did not differ between site types (Fig. 1). The Tukey post-hoc test did not detect a significant difference in evenness among groups. Shannon diversity index, percent EPT, percent Chironomidae, clinger taxa richness, clinger abundance, Trichoptera taxa richness, and Trichoptera abundance did not differ between site types, student activity periods, or exhibit a significant interaction effect within riffles (Table 2).

**Table 1:** Number (percent) of each macroinvertebrate taxa captured in riffles, runs, and pools within the class site and unused site in Alum Creek, Ohio, February 2013 to January 2014.

Taxa	Habit Guild	Riffle	Run	Pool
Chironomidae	Burrowers	**11459 (52.3)**	**11934 (80.8)**	**12488 (80.2)**
Hydropsychidae	Clingers	**2376 (10.8)**	156 (1.1)	5 (<1.0)
Turbellaria		**1390 (6.3)**	215 (1.5)	121 (<1.0)
Elmidae	Clingers	**1301 (5.9)**	**483 (3.3)**	**154 (<1.0)**
Simuliidae	Clingers	**1132 (5.2)**	11 (<1.0)	5 (<1.0)
Capniidae	Sprawlers	1072 (4.9)	**569 (3.9)**	27 (<1.0)
Heptageniidae	Clingers	965 (4.4)	**271 (1.8)**	**173 (1.1)**
Baetidae	Swimmers	433 (2.0)	136 (<1.0)	64 (<1.0)
Philopotamidae	Clingers	392 (1.8)	27 (<1.0)	0 (<1.0)
Psephenidae	Clingers	358 (1.6)	61 (<1.0)	18 (<1.0)
Caenidae	Sprawlers	263 (1.2)	**450 (3.1)**	**1550 (10.0)**
Pleuroceridae		101 (<1.0)	62 (<1.0)	95 (<1.0)
Sphaeriidae		93 (<1.0)	16 (<1.0)	41 (<1.0)
Tipulidae	Burrowers	83 (<1.0)	40 (<1.0)	12 (<1.0)
Ceratopogonidae	Sprawlers	76 (<1.0)	73 (<1.0)	**427 (2.7)**
Leuctridae	Sprawlers	59 (<1.0)	14 (<1.0)	0 (0)
Coenegrionidae	Climbers	41 (<1.0)	30 (<1.0)	10 (<1.0)
Isonychiidae	Swimmers	30 (<1.0)	4 (<1.0)	0 (0)
Empididae	Sprawlers	28 (<1.0)	14 (<1.0)	2 (<1.0)
Leptophlebiidae	Swimmers	26 (<1.0)	33 (<1.0)	41 (<1.0)
Perlidae	Clingers	20 (<1.0)	8 (<1.0)	0 (0)
Helicopsychidae	Clingers	18 (<1.0)	14 (<1.0)	20 (<1.0)
Ancylidae		17 (<1.0)	7 (<1.0)	61 (<1.0)
Asellidae		16 (<1.0)	29 (<1.0)	28 (<1.0)
Athericidae	Burrowers	16 (<1.0)	1 (<1.0)	2 (<1.0)
Acariformes		15 (<1.0)	7 (<1.0)	22 (<1.0)
Chloroperlidae	Clingers	15 (<1.0)	2 (<1.0)	0 (0)
Collembola	Sprawlers	12 (<1.0)	10 (<1.0)	15 (<1.0)
Gomphidae	Burrowers	11 (<1.0)	21 (<1.0)	14 (<1.0)
Nemouridae	Sprawlers	10 (<1.0)	6 (<1.0)	0 (0)
Cambaridae		8 (<1.0)	3 (<1.0)	3 (<1.0)
Ephemerellidae	Clingers	7 (<1.0)	17 (<1.0)	27 (<1.0)
Gerridae	Skaters	7 (<1.0)	2 (<1.0)	39 (<1.0)
Corydalidae	Clingers	6 (<1.0)	5 (<1.0)	2 (<1.0)
Polycentropodidae	Clingers	6 (<1.0)	2 (<1.0)	0 (0)
Sialidae	Burrowers	5 (<1.0)	0 (0)	2 (<1.0)
Calopterygidae	Climbers	4 (<1.0)	0 (0)	5 (<1.0)
Ephemeridae	Burrowers	4 (<1.0)	8 (<1.0)	29 (<1.0)
Physidae		4 (<1.0)	2 (<1.0)	11 (<1.0)
Tabanidae	Sprawlers	4 (<1.0)	3 (<1.0)	18 (<1.0)
Uenoidae	Clingers	4 (<1.0)	0 (0)	0 (0)
Hydroptilidae	Clingers	3 (<1.0)	9 (<1.0)	0 (0)
Leptoceridae	Climbers	3 (<1.0)	5 (<1.0)	15 (<1.0)
Taenyopterigidae	Sprawlers	3 (<1.0)	0 (0)	0 (0)
Amelitidae	Swimmers	2 (<1.0)	0 (0)	0 (0)
Limnephilidae	Sprawlers	2 (<1.0)	2 (<1.0)	1 (<1.0)
Scirtidae	Climbers	2 (<1.0)	1 (<1.0)	4 (<1.0)
Amphipoda		1 (<1.0)	0 (0)	0 (0)
Hydrophilidae	Climbers	1 (<1.0)	0 (0)	2 (<1.0)
Lepidostomatidae	Climbers	1 (<1.0)	0 (0)	1 (<1.0)
Libellulidae	Sprawlers	1 (<1.0)	0 (0)	0 (0)
Perlodidae	Clingers	1 (<1.0)	2 (<1.0)	0 (0)
Psychodidae	Burrowers	1 (<1.0)	1 (<1.0)	6 (<1.0)
Psychomyiidae	Clingers	1 (<1.0)	0 (0)	0 (0)
Rhyacophilidae	Clingers	1 (<1.0)	1 (<1.0)	0 (0)
Veliidae	Skaters	1 (<1.0)	0 (0)	0 (0)
Corixidae	Swimmers	0 (0)	0 (0)	3 (<1.0)
Culicidae	Swimmers	0 (0)	2 (<1.0)	2 (<1.0)
Dolichopodidae	Burrowers	0 (0)	1 (<1.0)	1 (<1.0)
Dytiscidae	Swimmers	0 (0)	1 (<1.0)	1 (<1.0)
Haliplidae	Climbers	0 (0)	1 (<1.0)	6 (<1.0)
Hirudinea		0 (0)	0 (0)	1 (<1.0)

Numbers and percents in bold signify those taxa that were the five most abundant taxa within a habitat type. Habit guild assignments were made using information from Poff *et al*. (2006), Vieira *et al*. (2006), and Merritt *et al*. (2008).

**Table 2:** Data transformation used, random effect used, and *P* values from a two factor (site type and student activity period) linear mixed effect model analysis of macroinvertebrate community response variables in riffles within two sites in Alum Creek, Ohio from February 2013 to January 2014.

Response Variable	Transformation	Random effect	ST	SA	ST x SA
Abundance	log	~1|Month	0.153	0.351	**0.019**
Taxa richness	n	~1|Month	0.107	0.134	**0.007**
Shannon diversity index	log	~1|Month	0.154	0.509	0.772
Evenness	log	~1|Month	0.351	0.542	**0.015**
Percent EPT	n	~1|Month	0.785	0.516	0.384
Percent Chironomidae	n	~1|Month	0.069	0.959	0.255
Clinger taxa richness	n	~1|Month	0.314	0.056	0.086
Clinger abundance	log	~1|Month	0.206	0.117	0.162
Trichoptera taxa richness	n	~1|Month	0.072	0.151	0.125
Trichoptera abundance	log	~1|Month	0.064	0.216	0.206
NMS axis 1	n	~1|Month	0.127	0.767	0.143
NMS axis 2	n	~1|Month	0.831	0.153	0.578

Bolded *P* values are those that had a significant effect (*P* < 0.05) within the linear mixed effect model analyses. Abbreviations are as follows: ST – site type; SA – student activity period; log – log(x + 1); n – no transformation.

**Figure 2:**
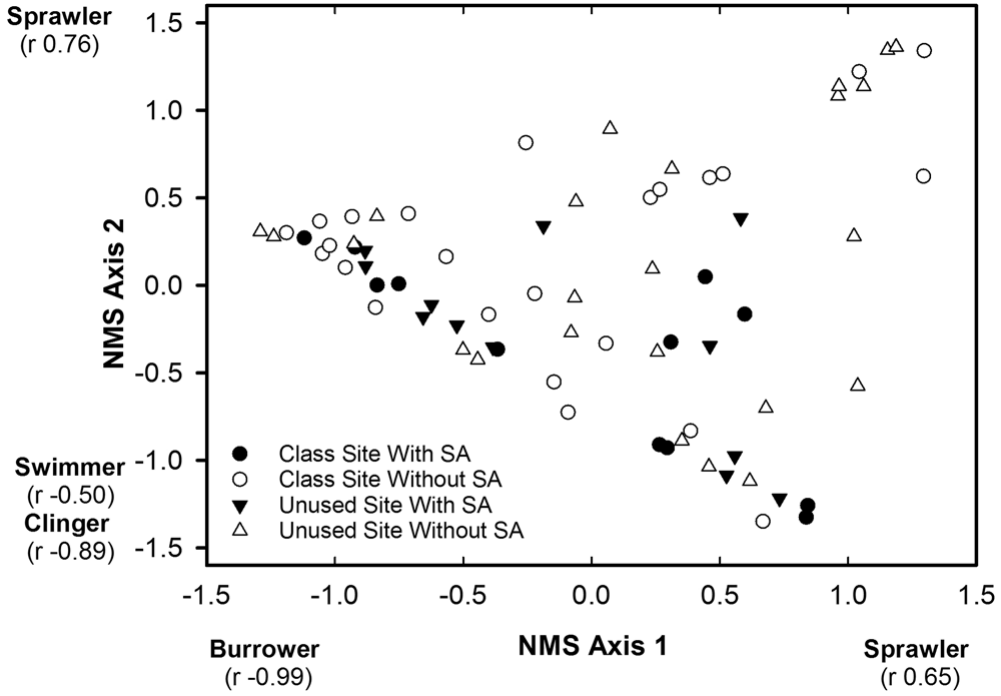
Site scores from the non-metric multidimensional scaling of burrowers, clingers, sprawlers, and swimmers within riffles of the class site and the unused site during time periods with and without student activity (SA), Alum Creek, Ohio, February 2013 to January 2014. Correlation coefficients (r) associated with each habit guild are the correlation coefficients from the correlation between the site scores and the percentage of each habit guild.

The NMS of habit guilds in riffles resulted in a two dimensional solution having a stress of 0.002, which indicates an excellent solution^40^. NMS axis 1 represented a gradient of sprawlers and burrowers where increasing site scores were positively correlated with sprawler relative abundance and negatively correlated with burrower relative abundance (Fig. 2). NMS axis 2 represented a gradient of clingers, swimmers, and sprawlers where increasing site scores exhibited positive correlation with sprawler relative abundance and negative correlations with the relative abundance of clingers and swimmers (Fig. 2). NMS axis 1 site scores and NMS axis 2 site scores did not differ between site types, student activity periods, or exhibit a significant interaction effect within riffles (Table 2), which indicated that habit guild composition within riffles was not influenced by site type or student activity period.

### Runs

**Figure 3:**
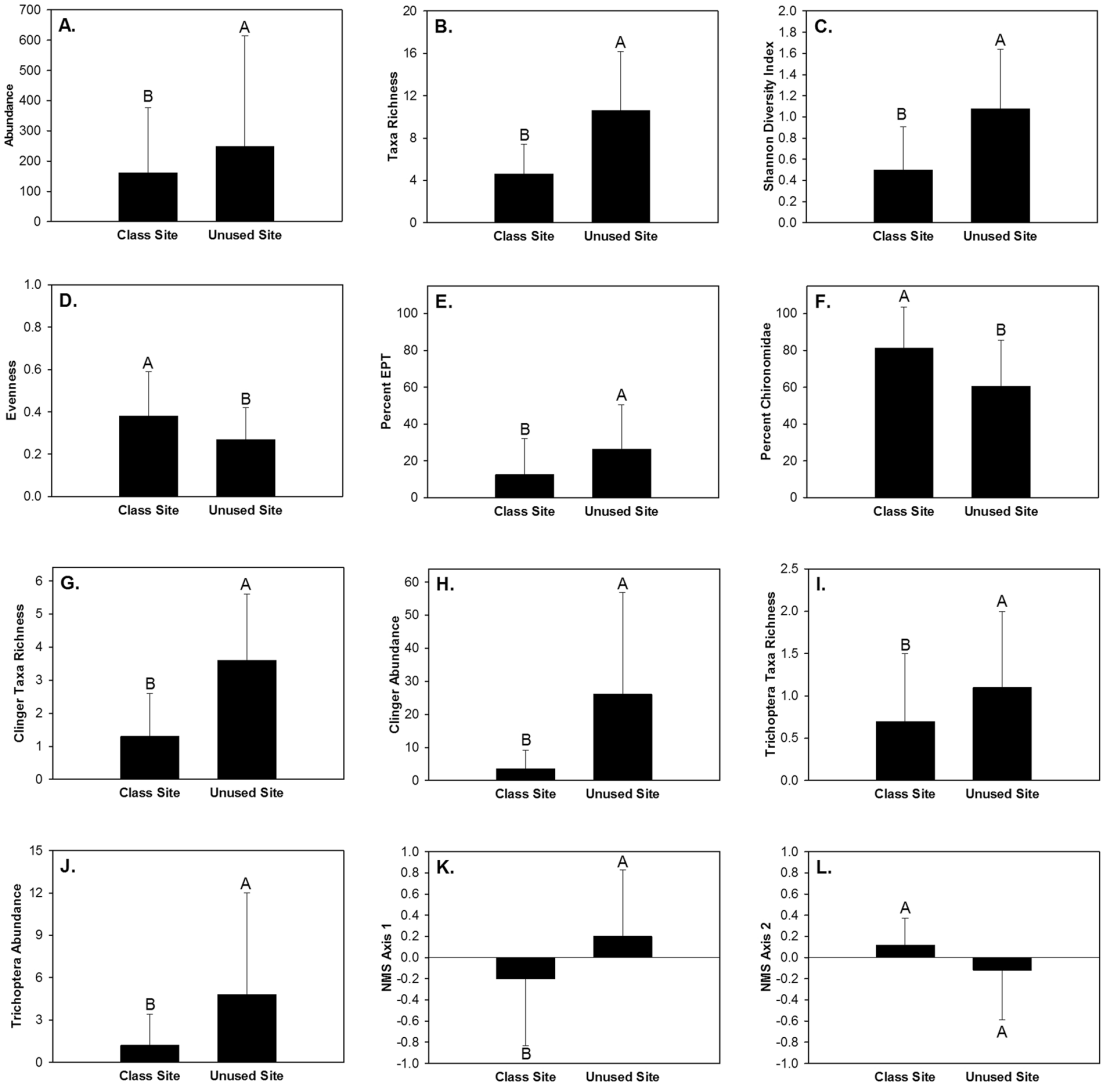
Mean and standard deviation of macroinvertebrate abundance (**A**), taxa richness (**B**), Shannon Diversity Index (**C**), evenness (**D**), percent Ephemeroptera, Plecoptera, Trichoptera (EPT) (**E**), percent Chironomidae (**F**), clinger taxa richness (**G**), clinger abundance (**H**), Trichoptera taxa richness (**I**), Trichoptera abundance (**J**), site scores of non-metric multidimensional scaling axis 1 (NMS1)(**K**), and site scores of non-metric multi-dimensional scaling axis 2 (NMS 2) (**L**) within runs in the class site and the unused site in Alum Creek, Ohio, February 2013 to January 2014.

**Figure 4:**
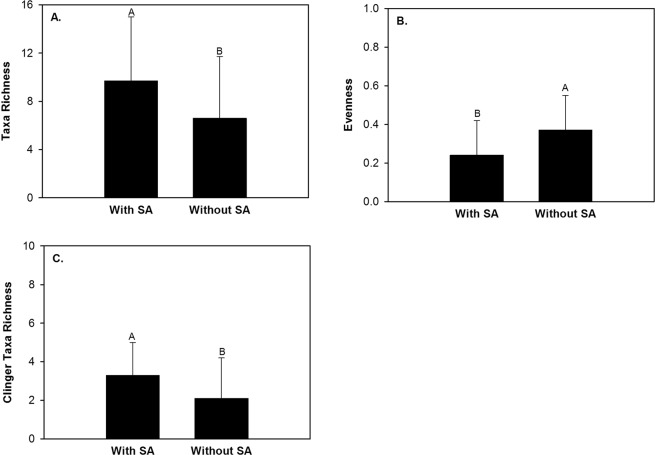
Mean and standard deviation of macroinvertebrate taxa richness (**A**), evenness (**B**), and clinger taxa richness (**C**) within runs of the class and unused sites during time periods with student activity (SA) and time periods without SA in Alum Creek, Ohio, February 2013 to January 2014.

Within runs 14,772 macroinvertebrates and 49 taxa were captured during the 12 month study period (Table 1). The five most abundant taxa constituted 92.8% of all captures in runs and included Chironomidae, Capniidae, Elmidae, Caenidae, and Heptageniidae (Table 1). Macroinvertebrate abundance, taxa richness, Shannon Diversity Index, evenness, percent EPT, percent Chironomidae, clinger taxa richness, clinger abundance, Trichoptera taxa richness, and Trichoptera abundance in runs differed between site types (Table 3). Macroinvertebrate abundance, taxa richness, Shannon Diversity Index, percent EPT, clinger taxa richness, clinger abundance, Trichoptera taxa richness, and Trichoptera abundance were greater in runs in the unused site than runs in the class site (Fig. 3). Percent Chironomidae and evenness were greater in runs in the class site than runs in the unused site (Fig. 3). Taxa richness, evenness, and clinger taxa richness differed between student activity periods (Table 3). Taxa richness and clinger taxa richness were greater during time periods with student activity than periods without student activity (Fig. 4). Evenness was greater during time periods without student activity than time periods with student activity (Fig. 4). None of the 10 univariate macroinvertebrate community response variables exhibited a significant interaction effect (Table 3).

**Table 3:** Data transformation used, random effect used, and *P* values from a two factor (site type and student activity period) linear mixed effect model analysis of macroinvertebrate community response variables in runs within two sites in Alum Creek, Ohio from February 2013 to January 2014. Bolded *P* values are those that had a significant effect (*P* < 0.05) within the linear mixed effect model analysis.

Response Variable	Transformation	Random effect	ST	SA	ST x SA
Abundance	log	~1|Month	**0.009**	0.173	0.415
Taxa richness	log	~1|Plot	**<0.001**	**0.001**	0.147
Shannon diversity index	log	~1|Month/Site/Plot	**0.005**	0.421	0.271
Evenness	log	~1|Month	**0.007**	**0.031**	0.895
Percent EPT	as	~1|Month	**0.002**	0.717	0.156
Percent Chironomidae	as	~1|Month	**<0.001**	0.829	0.085
Clinger taxa richness	log	~1|Plot	**<0.001**	**<0.001**	0.172
Clinger abundance	log	~1|Month/Site/Plot	**0.002**	0.055	0.791
Trichoptera taxa richness	log	~1|Plot	**0.034**	0.098	0.180
Trichoptera abundance	log	~1|Plot	**0.001**	0.704	0.203
NMS axis 1	log1.5	~1|Month	**<0.001**	0.921	0.243
NMS axis 2	log1.5	~1|Month/Site/Plot	**0.048**	0.762	0.662

Abbreviations are as follows: ST – site type; SA – student activity period; log – log(x + 1); as – arcsine squareroot; n – no transformation; log1.5 – log(x + 1.5).

**Figure 5:**
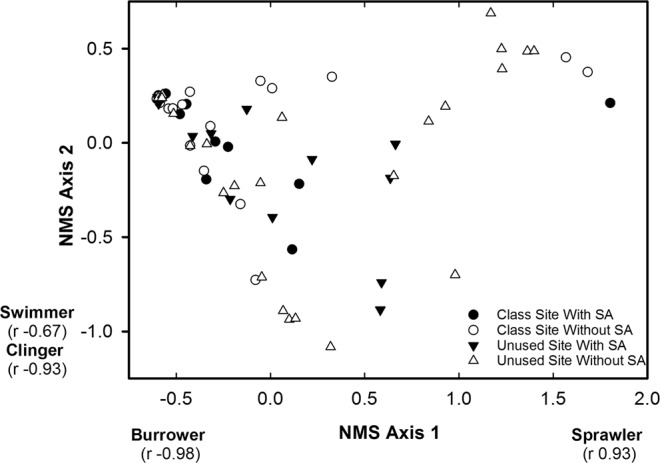
Site scores from the non-metric multidimensional scaling of burrowers, clingers, sprawlers, and swimmers within runs of the class site and the unused site during time periods with and without student activity (SA), Alum Creek, Ohio, February 2013 to January 2014. Correlation coefficients (r) associated with each habit guild are the correlation coefficients from the correlation between the site scores and the percentage of each habit guild.

The NMS of habit guilds in runs resulted in a two dimensional solution having a stress of 0.009, which indicates an excellent solution^40^. NMS axis 1 represented a gradient of sprawlers and burrowers where increasing site scores were positively correlated with the sprawler relative abundance and negatively correlated with burrower relative abundance (Fig. 5). NMS axis 2 represented a gradient of clingers and swimmers where increasing site scores exhibited negative correlations with the relative abundance of clingers and swimmers (Fig. 5). NMS axis 1 site scores and NMS axis 2 site scores differed between site types, but did not differ between student activity periods or exhibit a significant interaction effect within runs (Table 3). NMS axis 1 site scores were greater in runs within the unused site than runs within the class site. This result indicated that runs in the unused site had a greater relative abundance of sprawlers and runs in the class site had a greater relative abundance of burrowers (Fig. 3). The Tukey post hoc test did not detect a difference between NMS axis 2 site scores between runs within the class site and the unused site (Fig. 3).

### Pools

**Figure 6:**
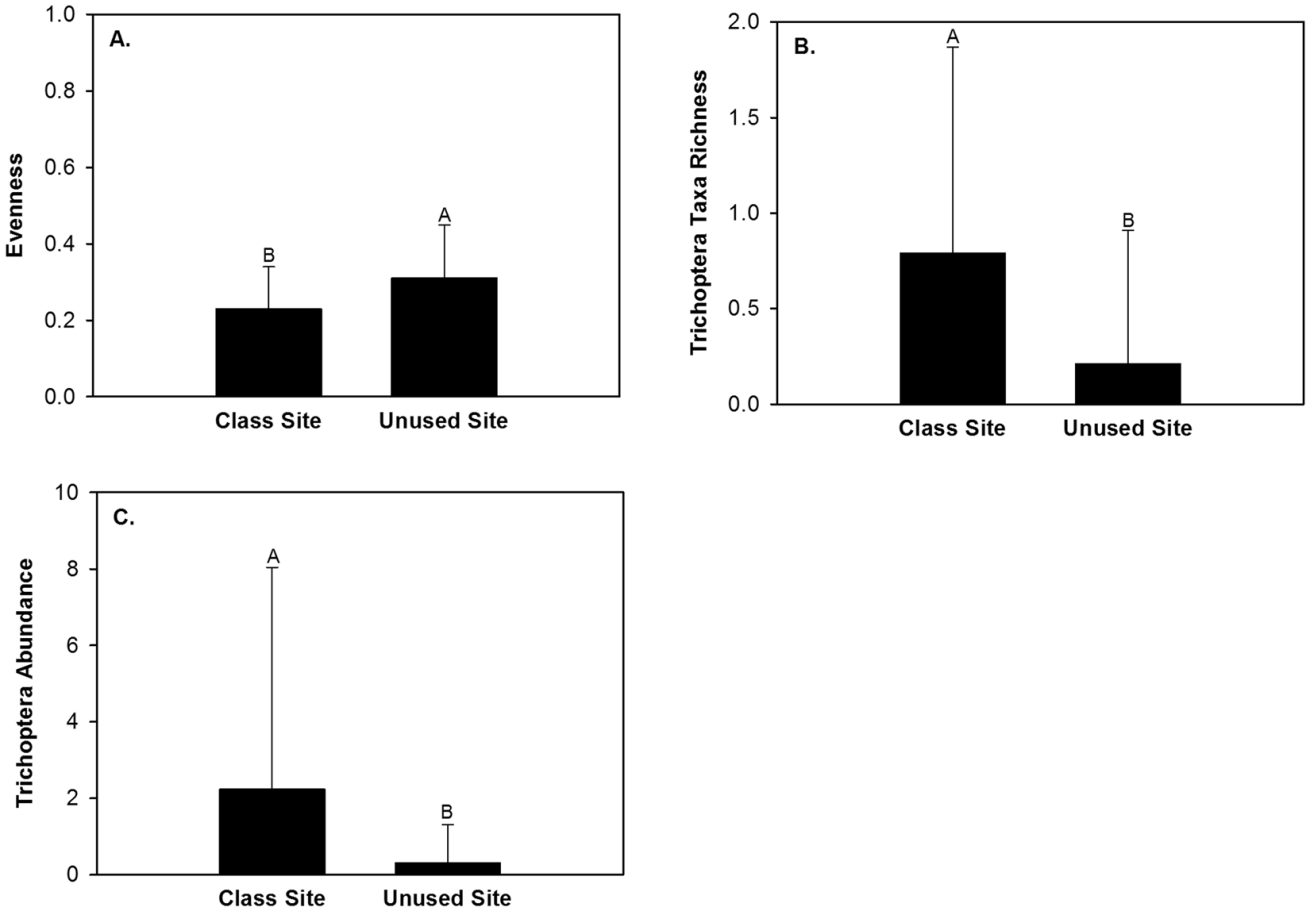
Mean and standard deviation of macroinvertebrate evenness (**A**), Trichoptera taxa richness (**B**), and Trichoptera abundance (**C**) within pools in the class site and the unused site in Alum Creek, Ohio, February 2013 to December 2013.

Within pools 15,574 macroinvertebrates and 45 taxa were captured in 11 months of sampling (Table 1). The five most common taxa constituted 94.7% of all captures and consisted of Chironomidae, Caenidae, Ceratopogonidae, Heptageniidae, and Elmidae (Table 1). Macroinvertebrate evenness, Trichoptera taxa richness, and Trichoptera abundance differed between site types (Table 4). Evenness was greater in pools in the unused site than pools in the class site (Fig. 6). Trichoptera taxa richness and Trichoptera abundance were greater in pools in the class site than pools in the unused site (Fig. 6). None of the 10 univariate macroinvertebrate community response variables differed among student activity periods or exhibited a significant interaction effect (Table 4).

**Table 4:** Data transformation used, random effect used, and *P* values from a two factor (site type and student activity period) linear mixed effect model analysis of macroinvertebrate community response variables in pools within two sites in Alum Creek, Ohio from February 2013 to December 2013.

Response Variable	Transformation	Random effect	ST	SA	ST x SA
Abundance	log	~1|Month/Site/Plot	0.369	0.372	0.728
Taxa richness	log	~1|Month	0.167	0.933	0.219
Shannon diversity index	log	~1|Month	0.429	0.692	0.505
Evenness	log	~1|Month	**0.012**	0.475	0.148
Percent EPT	as	~1|Month	0.101	0.976	0.123
Percent Chironomidae	as	~1|Month	0.214	0.750	0.763
Clinger taxa richness	log	~1|Month	0.137	0.922	0.423
Clinger abundance	log	~1|Month	0.212	0.702	0.508
Trichoptera taxa richness	log	~1|Month	**0.009**	0.915	0.824
Trichoptera abundance	log	~1|Month	**0.016**	0.720	0.692
NMS axis 1	log4	~1|Plot	0.702	0.262	0.986

Bolded *P* values are those that had a significant effect (*P* < 0.05) within the linear mixed effect model analysis. Abbreviations are as follows: ST – site type; SA – student activity period; log – log(x + 1); as – arcsine squareroot; n – no transformation; log4 – log(x + 4).

**Figure 7:**
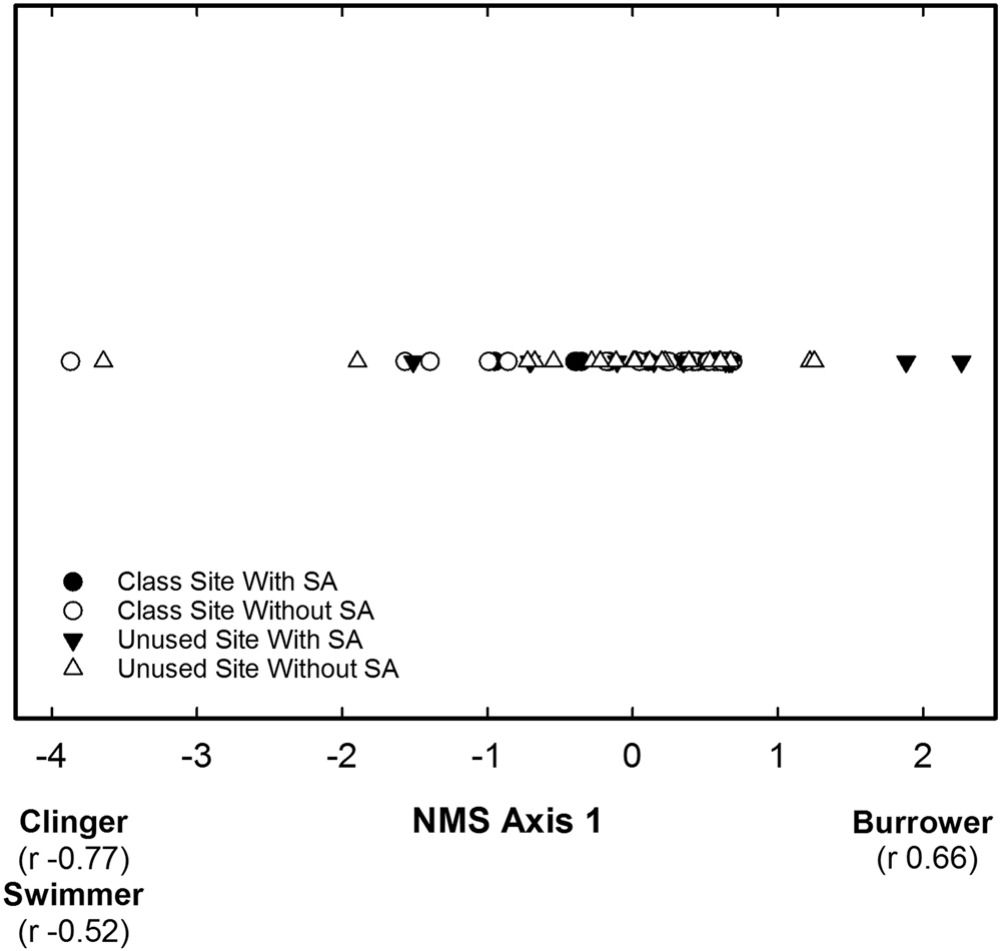
Site scores from the non-metric multidimensional scaling of burrowers, clingers, sprawlers, and swimmers within pools of the class site and the unused site during time periods with and without student activity (SA), Alum Creek, Ohio, February 2013 to December 2013. Correlation coefficients (r) associated with each habit guild are the correlation coefficients from the correlation between the site scores and the percentage of each habit guild.

The NMS of habit guilds in pools resulted in a one dimensional solution having a stress of 0.083, which indicates a good solution^40^. NMS axis 1 represented a gradient of burrowers, clingers, and swimmers where increasing site scores were positively correlated with burrower relative abundance and negatively correlated with the relative abundance of clingers and swimmers (Fig. 7). The site scores of NMS axis 1 did not differ between site types, student activity periods, or exhibit a significant interaction effect of site type and student activity period (Table 4), which indicated that habit guild composition in pools was not influenced by site type or student activity period.

## Discussion

The results did not support the hypothesis. ROE stream classes negatively impacted macroinvertebrate abundance and taxa richness only within riffles and not within runs and pools within a fourth order stream in central Ohio. Specifically, decreased macroinvertebrate abundance and taxa richness in riffles were observed at the class site compared to the unused site when stream classes were in session. No differences were observed between riffles from the class and unused sites when stream classes were not in session. Although statistically significant (P < 0.05) single factor effects of site type and student activity period within runs and pools were evident, these effects do not indicate the influence of ROE stream classes. The effect of site type on the response variable is the same in both student activity periods and the effect of student activity period on the response variable is the same in both site types^41^. The results from this study are consistent with those of previous studies^30,33,37–39^ that documented habitat-specific responses of macroinvertebrates to flooding and land use change. Others^30,33,37–39^ attributed their observed habitat-specific responses of macroinvertebrates to flooding and land use change to differences in taxa composition among habitat types and/or differences in physical habitat variables among habitat types. In contrast, the differential effect of ROE stream classes on macroinvertebrates in riffles as compared to runs and pools is attributed to the emphasis that is placed on the riffle during Heartland’s stream classes. The riffle is the focal point of the class site. Some Heartland instructors explicitly point out to students the likelihood of finding macroinvertebrates on rocks, and larger rocks in particular draw the attention of students. The students’ perception that large rocks host more macroinvertebrates along with the physical challenge posed by trying to move large rocks appears to concentrate student activity in the riffles^22^. Thus, the riffle in the class site with its numerous cobbles and larger sized rocks receives frequent student visitation. The riffle in the class site also serves as the entry and exit point for students into and out of the stream during ROE stream classes. As a result, the riffle at the class site is regularly trampled by students in transit while the runs and pools located on the opposite stream bank and upstream and downstream of the riffle receive comparatively less visitation from the students. These observations of student behavior suggest that the habitat-specific effect of ROE stream classes documented in this study depends in part on student behavior. If student activity is focused on one habitat type, then the effect of student trampling will be evident only in that habitat type. However, if student attention is directed to multiple habitat types, then it is possible that the effect of student trampling will span multiple habitat types. Future studies evaluating the impacts of recreational and educational activities on macroinvertebrates in streams need to explore whether and to what extent substrate disturbance resulting from these activities within a habitat type corresponds to the observed impact on macroinvertebrates.

The findings of decreased macroinvertebrate abundance and taxa richness within riffles as a result of ROE stream classes concurs with results from previous studies^21,22^ documenting the impact of ROE classes on macroinvertebrates in riffles. Notably, the previous results^21,22^ occurred within field studies conducted between April and May of 2014, which is after the sampling for this study was completed. This observation is important, because it suggests a multi-year impact of ROE stream classes on macroinvertebrates within the riffle of the class site in 2013 and 2014. The results within Alum Creek differ from an experimental simulation of student-induced trampling conducted in June of 2015 in the adjacent headwater tributaries. A one-time disturbance simulating the effect of trampling caused by ROE stream classes did not influence macroinvertebrate community structure in riffles of the tributaries^24^. These contrasting results are likely due to a combination of differences in the extent of site usage by ROE stream classes, substrate size, and stream size between the fourth order Alum Creek sites^this study,^^21,22^ and the first and second order tributary sites^24^. The contrasting results suggest that the ROE stream classes are more likely to affect macroinvertebrates in riffles that are more frequently visited by students, predominantly composed of cobble and other large substrate types, and located in fourth order streams. Conversely, ROE stream classes are less likely to affect macroinvertebrates in riffles that are less frequently visited by students, composed predominantly of gravel, and located in first or second order streams. Future research examining the influence of educational activities in streams needs to determine if the effect differs among streams with different substrate sizes and among different stream sizes.

These results documenting an impact of ROE stream classes on macroinvertebrates in riffles are similar to the results of other studies^13,17^ that documented a reduction in macroinvertebrate abundance and taxa richness in riffles in response to recreational activities. Laing^17^ observed that macroinvertebrate abundance and taxa richness were reduced in riffles within high use tributaries compared to riffles of lesser used tributaries of the Niobrara Scenic River in Nebraska. However, these reductions were confined to summer months when the riffles were subjected to high usage by canoers, kayakers, and tubers while no difference between stream types was evident during periods of low usage in spring and early autumn^17^. Hardiman and Burgin^13^ found that within a riffle in a fourth order upland canyon stream in Australia macroinvertebrate abundance and taxa richness were lower in experimentally trampled quadrats than untrampled quadrats immediately after experimental trampling was conducted. However, macroinvertebrate abundance and taxa richness recovered within 15 days after trampling within the experimentally trampled quadrats. Similarly, Holmquist *et al*.^20^ documented downstream impacts of recreational stream crossings and observed an increase in macroinvertebrate abundance and taxa richness below stream crossings compared to above the crossings in second orders streams in the Yosemite National Park, California. Heth *et al*.^19^ also documented increased taxa richness and EPT taxa richness downstream of recreational stream crossings in a Missouri stream in the summer. Our results from riffles and those of Laing^17^, Hardiman and Burgin^13^, Holmquist *et al*.^20^, and Heth *et al*.^19^ suggest that recreational and educational activities on macroinvertebrates in streams may have both localized and downstream impacts that influence macroinvertebrate abundance and taxa richness in different ways.

The results from runs and pools are similar to those studies^16,19,24^ that concluded that natural disturbances, among site differences, and temporal differences had a greater influence on macroinvertebrate community structure than recreational activities or ROE stream classes. Wright and Li^16^ documented that larval caddisfly density was lower in riffle and glide habitats in high use recreational sites compared to low use recreational sites in an Oregon stream before an 85 year event flood then did not differ between high and low use site types after the flood. Heth *et al*.^19^ found that macroinvertebrate communities within riffles in a Missouri River exhibited larger changes among sites than changes that occurred above and below stream crossings. Results from riffles in headwater tributaries within the Heartland property adjacent to Alum Creek^24^ documented that macroinvertebrate community structure was not influenced by experimental substrate disturbance, but differed among riffles within a stream and among time periods. The results from runs and pools combined with those of Wright and Li^16^, Heth *et al*.^19^, and Bossley and Smiley^24^ suggest that future research needs to identify the disturbance threshold that results in recreational and educational activities having a greater effect on macroinvertebrate communities than natural disturbances and/or spatio-temporal differences.

## Conclusions

In conclusion, the effects of student activity resulting from outdoor education stream classes on macroinvertebrate abundance and taxa richness were observed in riffles but not on other response variables or in other habitat types (i.e., runs, pools) during this year long study. These findings carry important implications for environmental education organizations that conduct stream classes, especially for those who serve large clienteles and seasonally or annually conduct numerous visits to the same stream site. Conducted over the long-term, heavy visitation at a stream site can negatively impact the stream biota, which can compromise the educational quality of the experience for participants. An underlying goal of many environmental education programs and outdoor education excursions is to provide students with a positive association with nature^23^. Stream classes achieve this goal by giving students an opportunity to capture and study the unique organisms that can be found in streams. The greater the variety and abundance of macroinvertebrates that live within a study reach, the greater the opportunity for discovery. Although some parks that regularly host recreationists have adopted the precautionary principle (i.e., assume the potential for harm until proven otherwise)^13^, we have observed that ROE organizations generally do not follow this practice. Based on the results of this study, we recommend that environmental education organizations maintain multiple sites within a stream designated for educational programming and alternate use between sites to avoid a reduction in macroinvertebrate abundance and taxa richness that might result from overuse of a single site.

## Methods

### Study locations and experimental design

The study was conducted at two sites within the upper Alum Creek, which is a fourth-order stream located in Morrow County in central Ohio, USA. The class site (40° 23′ 01.014″ N, 82° 52′ 37.870″ W) is a 61 m reach that has been regularly used since 2010 for stream classes taught by Heartland, which is an ROE center that provides overnight, multi-day, hands-on nature and environmental science programs for K–12 students. Heartland’s stream classes include measurements of select water chemistry variables, collection of live aquatic macroinvertebrates by dipnet, kick seine, and rock-picking, and on-site identification of captured organisms. During the collection of macroinvertebrates, students intentionally disturb the stream substrate by plowing into the gravel with their feet or flipping rocks over with their hands or feet. This process dislodges macroinvertebrates so they can be caught in nets. Students may also temporarily remove individual rocks from the water to pick off macroinvertebrates by hand. Macroinvertebrates captured by the students are released after identification. The class site is characterized by forested riparian habitat with riparian widths >30.5 m and bordered by an agricultural field on the right bank. Alum Creek at the class site is a C-4 riffle-pool stream^42^ with moderate sinuosity (=1.3), no constrictions, minimal to no canopy cover, and substrate composed primarily of coarse gravel (D50 = 38% coarse gravel substrate). The class site experiences more substrate disturbance and movement than the unused site due to the activity of ROE stream classes^22^. The unused site (40° 23′ 23.608″ N, 82° 52′ 27.751″ W) is a 61 m reach that served as the control and has never been used for stream classes by Heartland or other ROE organizations. The unused site is located approximately 1.2 km upstream of the class site. The unused site is also characterized by forested riparian habitat with riparian widths >30.5 m and an agricultural field bordering the riparian habitat on the right bank. Alum Creek at the unused site is a C-4 riffle-pool stream^42^ with moderate sinuosity (=1.3), no constrictions, minimal to no canopy cover, and substrate composed primarily of coarse gravel (D50 = 36% coarse gravel substrate) with some large boulders.

During the study water temperature, conductivity, total dissolved solids, turbidity, and nitrate concentrations were similar between the two sites (Table 5). The pH was greater in the upstream unused site than the class site, but both mean values indicated slightly basic conditions and only differed by 0.4 pH units (Table 5). From February 7, 2013 to January 4, 2014 mean daily discharge at the nearest USGS gauge 5.6 km downstream of the class site ranged from 0.08 to 82.9 m^3^/s with an annual average daily discharge of 2.4 m^3^/s. Mean daily discharge on sampling days ranged from 0.12 to 3.9 m^3^/s with an average of 0.8 m^3^/s, which confirmed our sampling was conducted under base flow conditions.

**Table 5:** Mean (standard deviation) of water chemistry variables measured in the class and unused sites in Alum Creek, Ohio from March 2013 to January 2014.

Response Variable	Class Site	Unused Site
Water temperature (°C)	9.7 (8.0) a	11.2 (8.0) a
pH	7.8 (0.3) a	8.2 (0.2) b
Conductivity (microsiemens)	746.7 (83.0) a	746.4 (115.4) a
Total dissolved solids (mg/L)	522.3 (56.0) a	520.2 (80.3) a
Turbidity (cm)	108.5 (22.7) a	106.8 (21.4) a
Nitrate (mg/L)	1.5 (2.6) a	0.9 (1.4) a

Different letters within a row indicate a significant difference (*P* < 0.05) in means as detected by linear mixed effect model analyses and subsequent Tukey post hoc tests.

The study was conducted from February 7, 2013 to January 4, 2014 and used a modified BACI (Before-After-Control-Impact) design^43,44^. The experimental design is a modification of the BACI design in that we sampled a control site (unused site) and a treatment site (class site) before and after two time periods with student activity during which stream classes were regularly conducted in the class site. The first time period with student activity extended from mid-April to early June 2013 and the second time period with student activity occurred from late September through early November 2013. Approximately 3500 students participated in 166 stream classes at the class site during the study. Specifically, 89 stream classes were conducted in the first student activity period and 77 stream classes were held in the second student activity period. Thus, these two time periods are characterized by repetitive pulse disturbances. Three time periods with no student activity from ROE stream classes (recovery periods) also occurred before and after both student activity time periods. The first recovery period occurred from February through mid-April 2013. The second recovery period occurred from early June through late September 2013 and the third recovery period occurred from early November 2013 through January 2014.

### Macroinvertebrate sampling

Macroinvertebrate sampling was conducted during the first week of each month from February 2013 to January 2014. Monthly macroinvertebrate sampling was conducted at the downstream class site first and then the upstream unused site. Three samples were collected each month from each of the three habitat types (riffle, run, pool) in the class site and the unused site. Each month we collected three Surber samples (500 μm net; 30.48 cm × 30.48 cm frame) from randomly selected locations in the single riffle at each site. The substrate within the frame of the Surber sampler was disturbed and mixed with a hand trowel to a depth of 10 cm or to the underlying bedrock for 30 seconds.

Each site included one run above the riffle and one run below the riffle. Thus, three Surber samples were collected from one run in each site during each month with sampling alternating between the lower and upper runs each month. Alternate sampling between lower and upper runs avoided the potential for compounding effects of investigator-induced disturbance on top of the student-induced disturbance^45^. Specifically, the lower run was sampled in February 2013 and all other even-numbered months (i.e., 4, 6, 8, 10, 12) throughout the study, while the upper run was sampled in March 2013 and all other odd-numbered months (i.e., 5, 7, 9, 11, 1) throughout the study. Substrate mixing as part of the Surber sampling in runs was conducted in the same manner as that used for the Surber sampling in riffles.

In addition to runs, pools and areas of slow-moving water along the stream edge (collectively, pools) were also located upstream and downstream of the riffle in each site. Three samples were collected from the pools in each site during each month. Alternate sampling of the lower and upper pools each month avoided the potential for investigator-induced disturbance^45^. The lower pools were sampled in February 2013 and subsequent even-numbered months, while the upper pools were sampled in March 2013 and subsequent odd-numbered months. Macroinvertebrates from pools were sampled with a stove pipe sampler (i.e., a bottomless 18.93 L bucket, diameter −30.5 cm) because the Surber sampler is not suitable for collecting macroinvertebrates within the deeper and slower flowing water typically found in pool habitats. Substrate within the stovepipe sampler was disturbed and mixed with a hand trowel to a depth of 10 cm or to the bedrock layer for 30 seconds. Following substrate disturbance the water column within the stovepipe sampler was strained by dipping a 500 μm hand net into the stovepipe sampler and dragging it through the water for 10 seconds. This straining process was conducted three times to maximize the likelihood of collecting all macroinvertebrates dislodged during the 30-second disturbance.

Contents of each sample were rinsed in a bucket of water, poured through a 500 μm hand net, transferred into a plastic bag, and preserved with 100% ethanol. The Surber net and hand net were also inspected for any clinging specimens, which were hand-picked and added to the bag prior to taking the next sample. In the laboratory, each sample was transferred into a plastic container and preserved in 100% ethanol for long-term storage until identification could be completed. No subsampling was conducted such that all macroinvertebrates from each sample were hand-picked using a 50x Wild Heerbrugg dissection microscope and stored in vials containing 95% ethanol. All specimens from each sample were later identified to class, subclass, super order, and family using Merritt *et al*.^46^ and Voshell^47^. All macroinvertebrates, except Turbellaria, Collembola, Hirudinea, Acariformes, and Amphipoda were identified to family level using Merritt *et al*.^46^ and Voshell^47^. Taxa not identified to family level were identified using Merritt *et al*.^46^ and Voshell^47^ as follows: Turbellaria (class), Collembola (subclass), Hirudinea (subclass), Acariformes (super order), and Amphipoda (order). Organisms too small or damaged to identify to the specified taxonomic resolution stated above were excluded from statistical analyses. This taxonomic resolution was chosen because our initial analyses^21–24^ and others evaluating the impacts of recreational activities^13,18^ indicated that it is sufficient for the evaluation of the impacts of ROE stream classes.

### Statistical analysis

Ten univariate community response variables were calculated for each sample from each habitat type (riffle, run, pool) at each site during each month. Response variables included: 1) macroinvertebrate abundance (number of macroinvertebrates captured in each sample); 2) taxa richness (number of taxa represented in each sample); 3) Shannon diversity index^48^; 4) evenness (calculated as E1/D)^49^; 5) percent EPT (percentage of Ephemeroptera, Plecoptera, Trichoptera calculated as EPT abundance divided by macroinvertebrate abundance); 6) percent Chironomidae (calculated as Chironomidae abundance divided by macroinvertebrate abundance); 7) Clinger taxa richness (number of clinger taxa in each sample); 8) Clinger abundance (number of clingers captured in each sample); 9) Trichoptera taxa richness (number of Trichoptera families in each sample); and 10) Trichoptera abundance (number of Trichoptera captured in each sample). The habit guild (burrowers, climbers, clingers, skaters, sprawlers, swimmers) of each insect taxa was compiled with family level information from Poff *et al*.^50^, Vieira *et al*.^51^, and Merritt *et al*.^46^ (Table 1). Non-metric multidimensional scaling (NMS) was conducted for each habitat type with the percentages of burrowers, clingers, sprawlers, and swimmers. These ordination analyses yielded site scores from the first two NMS axes that describe the changes in habit guild composition that occurs in each habitat type between site types and student activity periods. Climbers and skaters were excluded from NMS of each habitat type because these habit guilds were rare (i.e., having relative abundance values <1% of all captures and values of percent occurrence from all samples <39%). NMS was conducted with PC-ORD^52^ using the Sorensen (Bray Curtis) distance matrix and 500 iterations.

We used linear mixed effect model analysis with site type (class site, unused site) and student activity period (time periods with and without student activity) as fixed effects to determine if macroinvertebrate community response variables differed among site types and student activity periods. Linear mixed effect model analyses were conducted because they enable us to address the effect of pseudoreplication that occurs as a result of repeatedly sampling the same sites through time by incorporating a random effect into the model. The random effect used in each linear mixed effect model was that which was identified as the best random effect (Tables 2–4) from preliminary evaluations with the Akaike’s Information Criteria (AIC) from among nine possible random effects. The nine possible random effects included: 1) ~1|site type; 2) ~1|month; 3) ~1|plot (individual sample); 4) ~1|site type/plot; 5) ~1|month/plot; 6) ~1|month/site type/plot; 7) ~month|site type; 8) ~month|plot; 9) ~month|site type/plot. Linear mixed effect model analyses were conducted for each habitat type (riffle, run, pool) separately. As such the analyses consisted of 12 linear mixed effect model analyses for each habitat type (36 total). The focus with these linear mixed effect model analyses was the detection of significant interaction effects of site type and student activity period. This was crucial because the detection of effects in before-after-control-impact designs depends on observing a change in response variable trends between the control and treatment sites (i.e., between site types) following an impact (i.e., periods with student activity)^41^.

Linear mixed effect model analyses and AIC tests were conducted with the lme function in the nlme package^53^. The Tukey Test (lsmeans function, emmeans package)^54^ was used to determine differences among means if the linear mixed effect model analyses indicated that a significant effect occurred. Prior to conducting linear mixed effect model analyses we examined normal q-q plots created with the qqPlot function in the car package^55^, conducted the Shapiro-Wilk normality test using the shapiro.test function^56^, and conducted the Levene’s test for homogeneity of variance with the leveneTest function in the car package^55^ to determine if the response variables met the assumptions of normality and equal variance. Response variables that did not meet the assumptions were either log transformed or arcsine square root transformed prior to linear mixed effect model analyses (Tables 2–4). Univariate community response variables were log(x + 1) transformed, but negative values with some of the multivariate NMS site scores required the use of either log(x + 1.5) or log(x + 4) transformations (Tables 3, 4). Linear mixed effect model analyses, Tukey Tests, normal q-q plots, Shapiro-Wilk normality tests and Levene’s tests for homogeneity of variances were conducted with R^56^ and a significance level of 0.05.

## Supplementary information

Supplementary Information Tables A1-A6

## Data Availability

All data generated or analyzed during this study are included in this published article and its supplementary information files.
