# Supplementary material for: Impact of student-induced disturbance on stream macroinvertebrates differs among habitat types
Source: Sci Rep. 2019 Feb 5;9:1447. doi: 10.1038/s41598-018-38210-1 (PMC6363764; doi:10.1038/s41598-018-38210-1)
Supplement: Supplementary file 1 — Supplementary Information Tables A1-A6 [file 41598_2018_38210_MOESM1_ESM.docx]

**Supplementary Information for Jon P. Bossley & Peter. C. Smiley Jr. Impact of student-induced disturbance on stream macroinvertebrates differs among habitat types**

**Table A1** Univariate response variables from samples collected from riffles within a class site (1Exp) and an unused site (2Con) within Alum Creek, Ohio from February 2013 to January 2014.

| **Month** | **Year** | **SiteTrt** | **SA period** | **Plot** | **Sample#** | **Abundance** | **Taxa Richness** | **Shannon Diversity Index** | **Evenness** | **Percent EPT** | **Percent Chironomidae** | **Clinger Taxa Richness** | **Clinger Abundance** | **Trichoptera Taxa Richness** | **Trichoptera Abundance** |
| --- | --- | --- | --- | --- | --- | --- | --- | --- | --- | --- | --- | --- | --- | --- | --- |
| February | 2013 | 1Exp | no | Fe1 | Riffle1 | 84 | 10 | 1.71 | 0.38 | 0.810 | 0.107 | 5 | 21 | 2 | 4 |
| February | 2013 | 1Exp | no | Fe2 | Riffle2 | 23 | 5 | 0.90 | 0.35 | 0.826 | 0.130 | 1 | 1 | 0 | 0 |
| February | 2013 | 1Exp | no | Fe3 | Riffle3 | 53 | 5 | 0.56 | 0.26 | 0.075 | 0.868 | 2 | 3 | 0 | 0 |
| February | 2013 | 2Con | no | Fe4 | Riffle1 | 58 | 6 | 0.85 | 0.29 | 0.793 | 0.172 | 2 | 2 | 1 | 1 |
| February | 2013 | 2Con | no | Fe5 | Riffle2 | 135 | 15 | 1.84 | 0.28 | 0.356 | 0.422 | 7 | 39 | 3 | 4 |
| February | 2013 | 2Con | no | Fe6 | Riffle3 | 25 | 5 | 0.85 | 0.34 | 0.800 | 0.120 | 1 | 1 | 0 | 0 |
| March | 2013 | 1Exp | no | 3M1 | Riffle1 | 53 | 11 | 1.61 | 0.26 | 0.283 | 0.566 | 6 | 13 | 2 | 5 |
| March | 2013 | 1Exp | no | 3M2 | Riffle2 | 145 | 6 | 0.80 | 0.26 | 0.207 | 0.786 | 2 | 11 | 0 | 0 |
| March | 2013 | 1Exp | no | 3M3 | Riffle3 | 67 | 6 | 0.96 | 0.35 | 0.343 | 0.612 | 2 | 2 | 1 | 1 |
| March | 2013 | 2Con | no | 3M4 | Riffle1 | 50 | 8 | 1.43 | 0.36 | 0.700 | 0.180 | 3 | 4 | 0 | 0 |
| March | 2013 | 2Con | no | 3M5 | Riffle2 | 92 | 15 | 2.21 | 0.44 | 0.620 | 0.185 | 6 | 28 | 3 | 10 |
| March | 2013 | 2Con | no | 3M6 | Riffle3 | 43 | 7 | 1.52 | 0.51 | 0.605 | 0.233 | 1 | 3 | 0 | 0 |
| April | 2013 | 1Exp | no | Ap1 | Riffle1 | 791 | 12 | 0.53 | 0.11 | 0.032 | 0.880 | 5 | 81 | 3 | 5 |
| April | 2013 | 1Exp | no | Ap2 | Riffle2 | 749 | 20 | 0.68 | 0.07 | 0.055 | 0.873 | 7 | 64 | 3 | 17 |
| April | 2013 | 1Exp | no | Ap3 | Riffle3 | 655 | 14 | 0.50 | 0.09 | 0.081 | 0.902 | 5 | 24 | 1 | 2 |
| April | 2013 | 2Con | no | Ap4 | Riffle1 | 279 | 17 | 1.72 | 0.20 | 0.122 | 0.491 | 7 | 95 | 2 | 15 |
| April | 2013 | 2Con | no | Ap5 | Riffle2 | 206 | 13 | 0.80 | 0.11 | 0.078 | 0.840 | 7 | 21 | 3 | 4 |
| April | 2013 | 2Con | no | Ap6 | Riffle3 | 276 | 15 | 0.89 | 0.10 | 0.094 | 0.815 | 5 | 18 | 2 | 3 |
| May | 2013 | 1Exp | yes | 5M1 | Riffle1 | 182 | 16 | 1.15 | 0.11 | 0.110 | 0.758 | 7 | 32 | 2 | 6 |
| May | 2013 | 1Exp | yes | 5M2 | Riffle2 | 28 | 4 | 0.56 | 0.34 | 0.036 | 0.857 | 2 | 3 | 0 | 0 |
| May | 2013 | 1Exp | yes | 5M3 | Riffle3 | 615 | 10 | 0.43 | 0.12 | 0.050 | 0.919 | 5 | 39 | 1 | 5 |
| May | 2013 | 2Con | yes | 5M4 | Riffle1 | 585 | 23 | 0.93 | 0.07 | 0.062 | 0.815 | 10 | 77 | 4 | 10 |
| May | 2013 | 2Con | yes | 5M5 | Riffle2 | 916 | 22 | 0.90 | 0.07 | 0.083 | 0.818 | 9 | 95 | 3 | 6 |
| May | 2013 | 2Con | yes | 5M6 | Riffle3 | 199 | 16 | 1.25 | 0.13 | 0.075 | 0.688 | 7 | 45 | 3 | 6 |
| continued |  |  |  |  |  |  |  |  |  |  |  |  |  |  |  |
| **Month** | **Year** | **SiteTrt** | **SA period** | **Plot** | **Sample#** | **Abundance** | **Taxa Richness** | **Shannon Diversity Index** | **Evenness** | **Percent EPT** | **Percent Chironomidae** | **Clinger Taxa Richness** | **Clinger Abundance** | **Trichoptera Taxa Richness** | **Trichoptera Abundance** |
| June | 2013 | 1Exp | yes | 6M1 | Riffle1 | 154 | 11 | 1.02 | 0.16 | 0.058 | 0.753 | 6 | 26 | 0 | 0 |
| June | 2013 | 1Exp | yes | 6M2 | Riffle2 | 218 | 12 | 1.50 | 0.28 | 0.106 | 0.353 | 5 | 132 | 1 | 18 |
| June | 2013 | 1Exp | yes | 6M3 | Riffle3 | 501 | 14 | 1.29 | 0.19 | 0.084 | 0.349 | 6 | 308 | 1 | 27 |
| June | 2013 | 2Con | yes | 6M4 | Riffle1 | 136 | 12 | 1.50 | 0.21 | 0.125 | 0.610 | 6 | 39 | 1 | 10 |
| June | 2013 | 2Con | yes | 6M5 | Riffle2 | 363 | 14 | 1.24 | 0.15 | 0.140 | 0.691 | 6 | 85 | 2 | 14 |
| June | 2013 | 2Con | yes | 6M6 | Riffle3 | 209 | 11 | 1.44 | 0.24 | 0.187 | 0.584 | 4 | 68 | 1 | 27 |
| July | 2013 | 1Exp | no | 7M1 | Riffle1 | 199 | 12 | 1.64 | 0.34 | 0.402 | 0.156 | 5 | 123 | 1 | 41 |
| July | 2013 | 1Exp | no | 7M2 | Riffle2 | 149 | 10 | 1.13 | 0.19 | 0.154 | 0.705 | 5 | 22 | 1 | 1 |
| July | 2013 | 1Exp | no | 7M3 | Riffle3 | 1079 | 22 | 0.37 | 0.05 | 0.028 | 0.942 | 8 | 39 | 3 | 19 |
| July | 2013 | 2Con | no | 7M4 | Riffle1 | 37 | 10 | 1.96 | 0.54 | 0.378 | 0.351 | 4 | 15 | 1 | 5 |
| July | 2013 | 2Con | no | 7M5 | Riffle2 | 143 | 13 | 2.15 | 0.59 | 0.434 | 0.126 | 5 | 63 | 2 | 22 |
| July | 2013 | 2Con | no | 7M6 | Riffle3 | 937 | 21 | 1.88 | 0.23 | 0.189 | 0.260 | 8 | 459 | 3 | 141 |
| August | 2013 | 1Exp | no | Au1 | Riffle1 | 157 | 14 | 1.98 | 0.40 | 0.484 | 0.287 | 5 | 78 | 1 | 38 |
| August | 2013 | 1Exp | no | Au2 | Riffle2 | 172 | 13 | 1.78 | 0.30 | 0.349 | 0.453 | 6 | 62 | 3 | 10 |
| August | 2013 | 1Exp | no | Au3 | Riffle3 | 39 | 10 | 1.47 | 0.26 | 0.282 | 0.590 | 4 | 10 | 1 | 1 |
| August | 2013 | 2Con | no | Au4 | Riffle1 | 67 | 4 | 0.39 | 0.30 | 0.015 | 0.910 | 1 | 2 | 0 | 0 |
| August | 2013 | 2Con | no | Au5 | Riffle2 | 971 | 11 | 1.78 | 0.47 | 0.188 | 0.197 | 5 | 578 | 1 | 130 |
| August | 2013 | 2Con | no | Au6 | Riffle3 | 766 | 18 | 2.00 | 0.34 | 0.304 | 0.210 | 6 | 388 | 2 | 171 |
| September | 2013 | 1Exp | no | Se1 | Riffle1 | 105 | 7 | 0.92 | 0.25 | 0.095 | 0.743 | 3 | 11 | 0 | 0 |
| September | 2013 | 1Exp | no | Se2 | Riffle2 | 331 | 17 | 1.74 | 0.23 | 0.456 | 0.417 | 5 | 121 | 1 | 70 |
| September | 2013 | 1Exp | no | Se3 | Riffle3 | 82 | 9 | 1.17 | 0.22 | 0.171 | 0.695 | 3 | 12 | 0 | 0 |
| September | 2013 | 2Con | no | Se4 | Riffle1 | 934 | 12 | 0.29 | 0.09 | 0.007 | 0.952 | 5 | 17 | 3 | 4 |
| September | 2013 | 2Con | no | Se5 | Riffle2 | 140 | 12 | 1.55 | 0.24 | 0.250 | 0.557 | 5 | 33 | 2 | 11 |
| September | 2013 | 2Con | no | Se6 | Riffle3 | 262 | 10 | 1.50 | 0.31 | 0.141 | 0.515 | 4 | 62 | 1 | 6 |
| October | 2013 | 1Exp | yes | Oc1 | Riffle1 | 122 | 10 | 1.84 | 0.50 | 0.607 | 0.139 | 6 | 87 | 2 | 32 |
| October | 2013 | 1Exp | yes | Oc2 | Riffle2 | 133 | 13 | 1.45 | 0.21 | 0.263 | 0.571 | 7 | 44 | 2 | 28 |
| October | 2013 | 1Exp | yes | Oc3 | Riffle3 | 160 | 16 | 1.99 | 0.33 | 0.631 | 0.125 | 6 | 118 | 3 | 51 |
| continued |  |  |  |  |  |  |  |  |  |  |  |  |  |  |  |
| **Month** | **Year** | **SiteTrt** | **SA period** | **Plot** | **Sample#** | **Abundance** | **Taxa Richness** | **Shannon Diversity Index** | **Evenness** | **Percent EPT** | **Percent Chironomidae** | **Clinger Taxa Richness** | **Clinger Abundance** | **Trichoptera Taxa Richness** | **Trichoptera Abundance** |
| October | 2013 | 2Con | yes | Oc4 | Riffle1 | 379 | 14 | 2.11 | 0.51 | 0.322 | 0.161 | 7 | 238 | 2 | 88 |
| October | 2013 | 2Con | yes | Oc5 | Riffle2 | 373 | 20 | 2.12 | 0.31 | 0.340 | 0.209 | 7 | 213 | 3 | 89 |
| October | 2013 | 2Con | yes | Oc6 | Riffle3 | 2170 | 20 | 1.62 | 0.17 | 0.594 | 0.245 | 7 | 1384 | 3 | 1154 |
| November | 2013 | 1Exp | yes | No1 | Riffle1 | 13 | 5 | 1.53 | 0.87 | 0.615 | 0.308 | 2 | 5 | 1 | 3 |
| November | 2013 | 1Exp | yes | No2 | Riffle2 | 230 | 15 | 1.80 | 0.27 | 0.513 | 0.387 | 5 | 95 | 2 | 20 |
| November | 2013 | 1Exp | yes | No3 | Riffle3 | 101 | 14 | 1.85 | 0.32 | 0.564 | 0.366 | 6 | 29 | 3 | 6 |
| November | 2013 | 2Con | yes | No4 | Riffle1 | 221 | 14 | 1.89 | 0.36 | 0.430 | 0.262 | 5 | 43 | 2 | 6 |
| November | 2013 | 2Con | yes | No5 | Riffle2 | 213 | 17 | 2.03 | 0.34 | 0.376 | 0.249 | 5 | 71 | 2 | 50 |
| November | 2013 | 2Con | yes | No6 | Riffle3 | 699 | 20 | 1.68 | 0.17 | 0.258 | 0.475 | 6 | 93 | 3 | 33 |
| December | 2013 | 1Exp | no | De1 | Riffle1 | 310 | 14 | 1.67 | 0.27 | 0.465 | 0.403 | 5 | 48 | 2 | 32 |
| December | 2013 | 1Exp | no | De2 | Riffle2 | 729 | 20 | 1.73 | 0.18 | 0.475 | 0.444 | 5 | 277 | 2 | 92 |
| December | 2013 | 1Exp | no | De3 | Riffle3 | 137 | 13 | 1.71 | 0.29 | 0.445 | 0.350 | 5 | 20 | 1 | 3 |
| December | 2013 | 2Con | no | De4 | Riffle1 | 622 | 15 | 1.69 | 0.25 | 0.359 | 0.460 | 5 | 168 | 2 | 141 |
| December | 2013 | 2Con | no | De5 | Riffle2 | 303 | 17 | 2.09 | 0.40 | 0.535 | 0.211 | 6 | 151 | 3 | 85 |
| December | 2013 | 2Con | no | De6 | Riffle3 | 276 | 11 | 1.12 | 0.20 | 0.696 | 0.221 | 5 | 19 | 1 | 11 |
| January | 2014 | 1Exp | no | Ja1 | Riffle1 | 56 | 9 | 1.56 | 0.39 | 0.589 | 0.357 | 4 | 9 | 2 | 7 |
| January | 2014 | 1Exp | no | Ja2 | Riffle2 | 56 | 9 | 1.56 | 0.39 | 0.589 | 0.339 | 2 | 5 | 1 | 4 |
| January | 2014 | 1Exp | no | Ja3 | Riffle3 | 41 | 9 | 1.58 | 0.39 | 0.488 | 0.415 | 4 | 6 | 1 | 1 |
| January | 2014 | 2Con | no | Ja4 | Riffle1 | 43 | 8 | 1.39 | 0.21 | 0.372 | 0.535 | 2 | 6 | 0 | 0 |
| January | 2014 | 2Con | no | Ja5 | Riffle2 | 7 | 3 | 1.00 | 0.86 | 0.571 | 0.429 | 1 | 1 | 0 | 0 |
| January | 2014 | 2Con | no | Ja6 | Riffle3 | 23 | 3 | 0.84 | 0.72 | 0.478 | 0.522 | 1 | 1 | 1 | 1 |

**Table A2** Site scores from the non-metric multidimensional scaling (NMS) axis 1 and axis 2 and the percent of each habit guild within individual samples from riffles within a class site (1Exp) and an unused site (2Con) within Alum Creek, Ohio from February 2013 to January 2014.

| **Month** | **Year** | **SiteTrt** | **SA period** | **Plot** | **Sample#** | **NMS Axis 1 site scores** | **NMS Axis 2 site scores** | **Percent Burrowers** | **Percent Climbers** | **Percent Clingers** | **Percent Skaters** | **Percent Sprawlers** | **Percent Swimmers** |
| --- | --- | --- | --- | --- | --- | --- | --- | --- | --- | --- | --- | --- | --- |
| February | 2013 | 1Exp | no | Fe1 | Riffle1 | 1.294 | 0.624 | 0.110 | 0.020 | 0.250 | 0.000 | 0.600 | 0.020 |
| February | 2013 | 1Exp | no | Fe2 | Riffle2 | 1.297 | 1.341 | 0.130 | 0.000 | 0.040 | 0.000 | 0.780 | 0.040 |
| February | 2013 | 1Exp | no | Fe3 | Riffle3 | -0.934 | 0.393 | 0.870 | 0.000 | 0.060 | 0.000 | 0.080 | 0.000 |
| February | 2013 | 2Con | no | Fe4 | Riffle1 | 1.153 | 1.343 | 0.180 | 0.000 | 0.040 | 0.000 | 0.790 | 0.000 |
| February | 2013 | 2Con | no | Fe5 | Riffle2 | 0.237 | 0.093 | 0.450 | 0.000 | 0.300 | 0.000 | 0.250 | 0.000 |
| February | 2013 | 2Con | no | Fe6 | Riffle3 | 1.187 | 1.362 | 0.170 | 0.000 | 0.040 | 0.000 | 0.790 | 0.000 |
| March | 2013 | 1Exp | no | 3M1 | Riffle1 | -0.221 | -0.048 | 0.570 | 0.040 | 0.250 | 0.000 | 0.110 | 0.040 |
| March | 2013 | 1Exp | no | 3M2 | Riffle2 | -0.714 | 0.410 | 0.790 | 0.000 | 0.080 | 0.000 | 0.130 | 0.010 |
| March | 2013 | 1Exp | no | 3M3 | Riffle3 | -0.256 | 0.815 | 0.630 | 0.000 | 0.030 | 0.000 | 0.340 | 0.000 |
| March | 2013 | 2Con | no | 3M4 | Riffle1 | 1.061 | 1.136 | 0.200 | 0.000 | 0.090 | 0.000 | 0.720 | 0.000 |
| March | 2013 | 2Con | no | 3M5 | Riffle2 | 1.023 | 0.280 | 0.200 | 0.000 | 0.330 | 0.000 | 0.470 | 0.010 |
| March | 2013 | 2Con | no | 3M6 | Riffle3 | 0.961 | 1.082 | 0.230 | 0.000 | 0.070 | 0.000 | 0.670 | 0.020 |
| April | 2013 | 1Exp | no | Ap1 | Riffle1 | -1.050 | 0.181 | 0.890 | 0.000 | 0.100 | 0.000 | 0.000 | 0.000 |
| April | 2013 | 1Exp | no | Ap2 | Riffle2 | -1.021 | 0.228 | 0.890 | 0.000 | 0.090 | 0.000 | 0.020 | 0.000 |
| April | 2013 | 1Exp | no | Ap3 | Riffle3 | -1.059 | 0.366 | 0.910 | 0.000 | 0.040 | 0.000 | 0.050 | 0.010 |
| April | 2013 | 2Con | no | Ap4 | Riffle1 | -0.080 | -0.270 | 0.530 | 0.000 | 0.370 | 0.000 | 0.090 | 0.000 |
| April | 2013 | 2Con | no | Ap5 | Riffle2 | -0.927 | 0.238 | 0.850 | 0.000 | 0.100 | 0.000 | 0.040 | 0.000 |
| April | 2013 | 2Con | no | Ap6 | Riffle3 | -0.837 | 0.394 | 0.830 | 0.010 | 0.070 | 0.000 | 0.100 | 0.000 |
| May | 2013 | 1Exp | yes | 5M1 | Riffle1 | -0.752 | 0.009 | 0.780 | 0.000 | 0.180 | 0.000 | 0.020 | 0.020 |
| May | 2013 | 1Exp | yes | 5M2 | Riffle2 | -0.923 | 0.218 | 0.860 | 0.000 | 0.110 | 0.000 | 0.040 | 0.000 |
| May | 2013 | 1Exp | yes | 5M3 | Riffle3 | -1.120 | 0.271 | 0.920 | 0.000 | 0.060 | 0.000 | 0.010 | 0.000 |
| May | 2013 | 2Con | yes | 5M4 | Riffle1 | -0.881 | 0.111 | 0.840 | 0.000 | 0.140 | 0.000 | 0.020 | 0.010 |
| May | 2013 | 2Con | yes | 5M5 | Riffle2 | -0.883 | 0.199 | 0.830 | 0.000 | 0.100 | 0.000 | 0.040 | 0.020 |
| May | 2013 | 2Con | yes | 5M6 | Riffle3 | -0.624 | -0.110 | 0.720 | 0.000 | 0.230 | 0.000 | 0.020 | 0.020 |
| June | 2013 | 1Exp | yes | 6M1 | Riffle1 | -0.836 | 0.002 | 0.810 | 0.000 | 0.180 | 0.000 | 0.000 | 0.010 |
| June | 2013 | 1Exp | yes | 6M2 | Riffle2 | 0.265 | -0.910 | 0.370 | 0.000 | 0.620 | 0.000 | 0.000 | 0.000 |
| continued |  |  |  |  |  |  |  |  |  |  |  |  |  |
| **Month** | **Year** | **SiteTrt** | **SA period** | **Plot** | **Sample#** | **NMS Axis 1 site scores** | **NMS Axis 2 site scores** | **Percent Burrowers** | **Percent Climbers** | **Percent Clingers** | **Percent Skaters** | **Percent Sprawlers** | **Percent Swimmers** |
| June | 2013 | 1Exp | yes | 6M3 | Riffle3 | 0.295 | -0.928 | 0.360 | 0.000 | 0.630 | 0.000 | 0.000 | 0.010 |
| June | 2013 | 2Con | yes | 6M4 | Riffle1 | -0.525 | -0.227 | 0.690 | 0.000 | 0.300 | 0.000 | 0.020 | 0.000 |
| June | 2013 | 2Con | yes | 6M5 | Riffle2 | -0.657 | -0.179 | 0.730 | 0.000 | 0.250 | 0.000 | 0.000 | 0.020 |
| June | 2013 | 2Con | yes | 6M6 | Riffle3 | -0.384 | -0.353 | 0.630 | 0.010 | 0.350 | 0.000 | 0.010 | 0.010 |
| July | 2013 | 1Exp | no | 7M1 | Riffle1 | 0.668 | -1.348 | 0.170 | 0.000 | 0.640 | 0.010 | 0.000 | 0.190 |
| July | 2013 | 1Exp | no | 7M2 | Riffle2 | -0.842 | -0.127 | 0.750 | 0.000 | 0.150 | 0.000 | 0.000 | 0.100 |
| July | 2013 | 1Exp | no | 7M3 | Riffle3 | -1.189 | 0.301 | 0.950 | 0.000 | 0.040 | 0.000 | 0.010 | 0.000 |
| July | 2013 | 2Con | no | 7M4 | Riffle1 | 0.256 | -0.380 | 0.420 | 0.000 | 0.450 | 0.000 | 0.120 | 0.000 |
| July | 2013 | 2Con | no | 7M5 | Riffle2 | 1.037 | -0.575 | 0.160 | 0.000 | 0.540 | 0.000 | 0.210 | 0.090 |
| July | 2013 | 2Con | no | 7M6 | Riffle3 | 0.352 | -0.887 | 0.340 | 0.000 | 0.620 | 0.000 | 0.020 | 0.020 |
| August | 2013 | 1Exp | no | Au1 | Riffle1 | 0.386 | -0.831 | 0.320 | 0.010 | 0.540 | 0.000 | 0.030 | 0.100 |
| August | 2013 | 1Exp | no | Au2 | Riffle2 | -0.147 | -0.552 | 0.500 | 0.010 | 0.380 | 0.000 | 0.020 | 0.100 |
| August | 2013 | 1Exp | no | Au3 | Riffle3 | -0.401 | -0.167 | 0.630 | 0.000 | 0.260 | 0.000 | 0.050 | 0.050 |
| August | 2013 | 2Con | no | Au4 | Riffle1 | -1.239 | 0.279 | 0.960 | 0.000 | 0.030 | 0.000 | 0.000 | 0.010 |
| August | 2013 | 2Con | no | Au5 | Riffle2 | 0.617 | -1.118 | 0.240 | 0.000 | 0.720 | 0.000 | 0.010 | 0.040 |
| August | 2013 | 2Con | no | Au6 | Riffle3 | 0.457 | -1.036 | 0.280 | 0.000 | 0.630 | 0.000 | 0.010 | 0.090 |
| September | 2013 | 1Exp | no | Se1 | Riffle1 | -0.960 | 0.101 | 0.850 | 0.000 | 0.120 | 0.010 | 0.000 | 0.020 |
| September | 2013 | 1Exp | no | Se2 | Riffle2 | -0.091 | -0.726 | 0.440 | 0.000 | 0.390 | 0.000 | 0.000 | 0.160 |
| September | 2013 | 1Exp | no | Se3 | Riffle3 | -0.567 | 0.164 | 0.710 | 0.000 | 0.150 | 0.030 | 0.090 | 0.030 |
| September | 2013 | 2Con | no | Se4 | Riffle1 | -1.291 | 0.308 | 0.980 | 0.000 | 0.020 | 0.000 | 0.000 | 0.000 |
| September | 2013 | 2Con | no | Se5 | Riffle2 | -0.501 | -0.369 | 0.610 | 0.020 | 0.250 | 0.000 | 0.010 | 0.120 |
| September | 2013 | 2Con | no | Se6 | Riffle3 | -0.444 | -0.424 | 0.570 | 0.040 | 0.260 | 0.000 | 0.010 | 0.120 |
| October | 2013 | 1Exp | yes | Oc1 | Riffle1 | 0.837 | -1.325 | 0.160 | 0.000 | 0.810 | 0.000 | 0.000 | 0.040 |
| October | 2013 | 1Exp | yes | Oc2 | Riffle2 | -0.366 | -0.365 | 0.620 | 0.000 | 0.350 | 0.000 | 0.010 | 0.020 |
| October | 2013 | 1Exp | yes | Oc3 | Riffle3 | 0.843 | -1.257 | 0.150 | 0.010 | 0.770 | 0.010 | 0.020 | 0.050 |
| October | 2013 | 2Con | yes | Oc4 | Riffle1 | 0.733 | -1.216 | 0.200 | 0.000 | 0.760 | 0.000 | 0.000 | 0.040 |
| October | 2013 | 2Con | yes | Oc5 | Riffle2 | 0.558 | -0.974 | 0.270 | 0.000 | 0.670 | 0.000 | 0.030 | 0.030 |
| October | 2013 | 2Con | yes | Oc6 | Riffle3 | 0.526 | -1.085 | 0.270 | 0.000 | 0.700 | 0.000 | 0.000 | 0.030 |
| continued |  |  |  |  |  |  |  |  |  |  |  |  |  |
| **Month** | **Year** | **SiteTrt** | **SA period** | **Plot** | **Sample#** | **NMS Axis 1 site scores** | **NMS Axis 2 site scores** | **Percent Burrowers** | **Percent Climbers** | **Percent Clingers** | **Percent Skaters** | **Percent Sprawlers** | **Percent Swimmers** |
| November | 2013 | 1Exp | yes | No1 | Riffle1 | 0.597 | -0.164 | 0.330 | 0.000 | 0.420 | 0.000 | 0.250 | 0.000 |
| November | 2013 | 1Exp | yes | No2 | Riffle2 | 0.309 | -0.324 | 0.400 | 0.020 | 0.430 | 0.000 | 0.140 | 0.010 |
| November | 2013 | 1Exp | yes | No3 | Riffle3 | 0.443 | 0.049 | 0.370 | 0.010 | 0.290 | 0.000 | 0.260 | 0.060 |
| November | 2013 | 2Con | yes | No4 | Riffle1 | 0.582 | 0.386 | 0.340 | 0.000 | 0.240 | 0.000 | 0.410 | 0.010 |
| November | 2013 | 2Con | yes | No5 | Riffle2 | 0.462 | -0.345 | 0.360 | 0.010 | 0.460 | 0.000 | 0.170 | 0.010 |
| November | 2013 | 2Con | yes | No6 | Riffle3 | -0.188 | 0.341 | 0.600 | 0.000 | 0.170 | 0.000 | 0.230 | 0.010 |
| December | 2013 | 1Exp | no | De1 | Riffle1 | 0.230 | 0.502 | 0.450 | 0.000 | 0.170 | 0.000 | 0.360 | 0.010 |
| December | 2013 | 1Exp | no | De2 | Riffle2 | 0.057 | -0.332 | 0.480 | 0.000 | 0.400 | 0.000 | 0.100 | 0.020 |
| December | 2013 | 1Exp | no | De3 | Riffle3 | 0.461 | 0.616 | 0.380 | 0.000 | 0.160 | 0.000 | 0.450 | 0.010 |
| December | 2013 | 2Con | no | De4 | Riffle1 | -0.066 | -0.070 | 0.540 | 0.000 | 0.310 | 0.000 | 0.140 | 0.000 |
| December | 2013 | 2Con | no | De5 | Riffle2 | 0.680 | -0.702 | 0.260 | 0.010 | 0.600 | 0.000 | 0.120 | 0.020 |
| December | 2013 | 2Con | no | De6 | Riffle3 | 0.964 | 1.138 | 0.230 | 0.000 | 0.070 | 0.000 | 0.690 | 0.000 |
| January | 2014 | 1Exp | no | Ja1 | Riffle1 | 0.512 | 0.637 | 0.360 | 0.020 | 0.160 | 0.000 | 0.450 | 0.000 |
| January | 2014 | 1Exp | no | Ja2 | Riffle2 | 1.043 | 1.221 | 0.210 | 0.000 | 0.060 | 0.000 | 0.730 | 0.000 |
| January | 2014 | 1Exp | no | Ja3 | Riffle3 | 0.266 | 0.547 | 0.440 | 0.000 | 0.150 | 0.000 | 0.380 | 0.030 |
| January | 2014 | 2Con | no | Ja4 | Riffle1 | -0.060 | 0.478 | 0.550 | 0.000 | 0.140 | 0.000 | 0.290 | 0.020 |
| January | 2014 | 2Con | no | Ja5 | Riffle2 | 0.313 | 0.665 | 0.430 | 0.000 | 0.140 | 0.000 | 0.430 | 0.000 |
| January | 2014 | 2Con | no | Ja6 | Riffle3 | 0.072 | 0.894 | 0.520 | 0.000 | 0.040 | 0.000 | 0.430 | 0.000 |

**Table A3** Univariate response variables from samples collected from runs within a class site (1Exp) and an unused site (2Con) within Alum Creek, Ohio from February 2013 to January 2014.

| **Month** | **Year** | **SiteTrt** | **SA period** | **Plot** | **Sample#** | **Abundance** | **Taxa Richness** | **Shannon Diversity Index** | **Evenness** | **Percent EPT** | **Percent Chironomidae** | **Clinger Taxa Richness** | **Clinger Abundance** | **Trichoptera Taxa Richness** | **Trichoptera Abundance** |
| --- | --- | --- | --- | --- | --- | --- | --- | --- | --- | --- | --- | --- | --- | --- | --- |
| February | 2013 | 1Exp | no | A | Run1 | 13 | 5 | 1.18 | 0.48 | 0.692 | 0.154 | 1 | 1 | 1 | 1 |
| February | 2013 | 1Exp | no | A | Run2 | 578 | 2 | 0.01 | 0.50 | 0.002 | 0.998 | 0 | 0 | 0 | 0 |
| February | 2013 | 1Exp | no | A | Run3 | 47 | 4 | 0.78 | 0.46 | 0.298 | 0.681 | 0 | 0 | 0 | 0 |
| February | 2013 | 2Con | no | A | Run1 | 258 | 2 | 0.03 | 0.50 | 0.004 | 0.996 | 1 | 1 | 1 | 1 |
| February | 2013 | 2Con | no | A | Run2 | 32 | 4 | 0.70 | 0.39 | 0.813 | 0.156 | 0 | 0 | 0 | 0 |
| February | 2013 | 2Con | no | A | Run3 | 49 | 9 | 1.66 | 0.44 | 0.490 | 0.388 | 6 | 16 | 2 | 2 |
| March | 2013 | 1Exp | no | B | Run1 | 560 | 5 | 0.05 | 0.20 | 0.005 | 0.993 | 3 | 3 | 1 | 1 |
| March | 2013 | 1Exp | no | B | Run2 | 8 | 2 | 0.38 | 0.64 | 0.000 | 0.875 | 0 | 0 | 0 | 0 |
| March | 2013 | 1Exp | no | B | Run3 | 32 | 2 | 0.23 | 0.57 | 0.063 | 0.938 | 0 | 0 | 0 | 0 |
| March | 2013 | 2Con | no | B | Run1 | 385 | 19 | 1.79 | 0.18 | 0.358 | 0.491 | 7 | 50 | 3 | 23 |
| March | 2013 | 2Con | no | B | Run2 | 227 | 19 | 1.85 | 0.23 | 0.626 | 0.225 | 7 | 12 | 2 | 4 |
| March | 2013 | 2Con | no | B | Run3 | 45 | 10 | 1.65 | 0.37 | 0.467 | 0.356 | 2 | 2 | 1 | 1 |
| April | 2013 | 1Exp | no | A | Run1 | 100 | 2 | 0.06 | 0.51 | 0.000 | 0.990 | 0 | 0 | 0 | 0 |
| April | 2013 | 1Exp | no | A | Run2 | 32 | 4 | 0.73 | 0.40 | 0.219 | 0.781 | 0 | 0 | 0 | 0 |
| April | 2013 | 1Exp | no | A | Run3 | 490 | 3 | 0.03 | 0.34 | 0.002 | 0.996 | 1 | 1 | 1 | 1 |
| April | 2013 | 2Con | no | A | Run1 | 104 | 3 | 0.15 | 0.35 | 0.019 | 0.971 | 0 | 0 | 0 | 0 |
| April | 2013 | 2Con | no | A | Run2 | 149 | 12 | 1.10 | 0.16 | 0.215 | 0.691 | 5 | 7 | 1 | 1 |
| April | 2013 | 2Con | no | A | Run3 | 160 | 15 | 1.22 | 0.13 | 0.188 | 0.706 | 5 | 24 | 1 | 1 |
| May | 2013 | 1Exp | yes | B | Run1 | 168 | 7 | 0.35 | 0.17 | 0.060 | 0.929 | 3 | 3 | 2 | 2 |
| May | 2013 | 1Exp | yes | B | Run2 | 615 | 7 | 0.14 | 0.15 | 0.013 | 0.979 | 2 | 5 | 2 | 5 |
| May | 2013 | 1Exp | yes | B | Run3 | 152 | 7 | 0.34 | 0.16 | 0.046 | 0.934 | 3 | 3 | 0 | 0 |
| May | 2013 | 2Con | yes | B | Run1 | 681 | 21 | 0.94 | 0.08 | 0.053 | 0.753 | 7 | 120 | 1 | 1 |
| May | 2013 | 2Con | yes | B | Run2 | 591 | 16 | 0.71 | 0.09 | 0.074 | 0.854 | 5 | 40 | 0 | 0 |
| May | 2013 | 2Con | yes | B | Run3 | 290 | 12 | 0.74 | 0.12 | 0.148 | 0.810 | 5 | 10 | 2 | 2 |
| June | 2013 | 1Exp | yes | A | Run1 | 19 | 4 | 0.83 | 0.44 | 0.000 | 0.737 | 1 | 3 | 0 | 0 |
| continued |  |  |  |  |  |  |  |  |  |  |  |  |  |  |  |
| **Month** | **Year** | **SiteTrt** | **SA period** | **Plot** | **Sample#** | **Abundance** | **Taxa Richness** | **Shannon Diversity Index** | **Evenness** | **Percent EPT** | **Percent Chironomidae** | **Clinger Taxa Richness** | **Clinger Abundance** | **Trichoptera Taxa Richness** | **Trichoptera Abundance** |
| June | 2013 | 1Exp | yes | A | Run2 | 36 | 6 | 0.71 | 0.24 | 0.083 | 0.833 | 2 | 3 | 1 | 2 |
| June | 2013 | 1Exp | yes | A | Run3 | 87 | 5 | 0.42 | 0.24 | 0.011 | 0.908 | 2 | 4 | 0 | 0 |
| June | 2013 | 2Con | yes | A | Run1 | 171 | 3 | 0.10 | 0.35 | 0.000 | 0.982 | 2 | 3 | 0 | 0 |
| June | 2013 | 2Con | yes | A | Run2 | 2170 | 7 | 0.07 | 0.15 | 0.006 | 0.991 | 3 | 8 | 1 | 2 |
| June | 2013 | 2Con | yes | A | Run3 | 305 | 7 | 0.55 | 0.18 | 0.069 | 0.879 | 4 | 22 | 1 | 4 |
| July | 2013 | 1Exp | no | B | Run1 | 34 | 4 | 0.71 | 0.39 | 0.059 | 0.794 | 2 | 3 | 1 | 2 |
| July | 2013 | 1Exp | no | B | Run2 | 3 | 2 | 0.64 | 0.90 | 0.000 | 0.667 | 1 | 1 | 0 | 0 |
| July | 2013 | 1Exp | no | B | Run3 | 94 | 4 | 0.22 | 0.27 | 0.011 | 0.957 | 1 | 1 | 1 | 1 |
| July | 2013 | 2Con | no | B | Run1 | 298 | 15 | 1.48 | 0.18 | 0.154 | 0.591 | 5 | 74 | 1 | 25 |
| July | 2013 | 2Con | no | B | Run2 | 324 | 13 | 1.12 | 0.14 | 0.068 | 0.719 | 4 | 48 | 1 | 6 |
| July | 2013 | 2Con | no | B | Run3 | 326 | 14 | 1.04 | 0.13 | 0.037 | 0.715 | 4 | 53 | 1 | 1 |
| August | 2013 | 1Exp | no | A | Run1 | 20 | 3 | 0.69 | 0.55 | 0.250 | 0.750 | 1 | 4 | 0 | 0 |
| August | 2013 | 1Exp | no | A | Run2 | 84 | 5 | 0.58 | 0.28 | 0.131 | 0.833 | 2 | 12 | 0 | 0 |
| August | 2013 | 1Exp | no | A | Run3 | 1 | 1 | 0.00 | 0.00 | 0.000 | 1.000 | 0 | 0 | 0 | 0 |
| August | 2013 | 2Con | no | A | Run1 | 12 | 3 | 0.72 | 0.56 | 0.000 | 0.750 | 1 | 1 | 0 | 0 |
| August | 2013 | 2Con | no | A | Run2 | 39 | 8 | 1.49 | 0.43 | 0.256 | 0.436 | 3 | 18 | 0 | 0 |
| August | 2013 | 2Con | no | A | Run3 | 137 | 14 | 1.01 | 0.11 | 0.066 | 0.788 | 3 | 12 | 0 | 0 |
| September | 2013 | 1Exp | no | B | Run1 | 63 | 4 | 0.24 | 0.28 | 0.032 | 0.952 | 2 | 2 | 1 | 1 |
| September | 2013 | 1Exp | no | B | Run2 | 397 | 2 | 0.02 | 0.50 | 0.003 | 0.997 | 0 | 0 | 0 | 0 |
| September | 2013 | 1Exp | no | B | Run3 | 839 | 3 | 0.05 | 0.34 | 0.006 | 0.993 | 0 | 0 | 0 | 0 |
| September | 2013 | 2Con | no | B | Run1 | 464 | 15 | 1.55 | 0.20 | 0.246 | 0.550 | 4 | 119 | 1 | 17 |
| September | 2013 | 2Con | no | B | Run2 | 150 | 11 | 1.57 | 0.30 | 0.307 | 0.507 | 4 | 52 | 1 | 17 |
| September | 2013 | 2Con | no | B | Run3 | 116 | 10 | 1.42 | 0.27 | 0.276 | 0.569 | 4 | 30 | 1 | 17 |
| October | 2013 | 1Exp | yes | A | Run1 | 207 | 12 | 0.90 | 0.13 | 0.145 | 0.802 | 5 | 18 | 3 | 5 |
| October | 2013 | 1Exp | yes | A | Run2 | 199 | 12 | 1.21 | 0.17 | 0.176 | 0.678 | 3 | 25 | 1 | 1 |
| October | 2013 | 1Exp | yes | A | Run3 | 58 | 11 | 1.42 | 0.22 | 0.328 | 0.621 | 4 | 12 | 2 | 2 |
| October | 2013 | 2Con | yes | A | Run1 | 28 | 7 | 1.19 | 0.32 | 0.214 | 0.643 | 3 | 3 | 0 | 0 |
| continued |  |  |  |  |  |  |  |  |  |  |  |  |  |  |  |
| **Month** | **Year** | **SiteTrt** | **SA period** | **Plot** | **Sample#** | **Abundance** | **Taxa Richness** | **Shannon Diversity Index** | **Evenness** | **Percent EPT** | **Percent Chironomidae** | **Clinger Taxa Richness** | **Clinger Abundance** | **Trichoptera Taxa Richness** | **Trichoptera Abundance** |
| October | 2013 | 2Con | yes | A | Run2 | 103 | 9 | 1.66 | 0.11 | 0.262 | 0.417 | 3 | 34 | 0 | 0 |
| October | 2013 | 2Con | yes | A | Run3 | 215 | 14 | 1.78 | 0.29 | 0.405 | 0.414 | 3 | 62 | 1 | 2 |
| November | 2013 | 1Exp | yes | B | Run1 | 10 | 2 | 0.33 | 0.61 | 0.100 | 0.900 | 1 | 1 | 0 | 0 |
| November | 2013 | 1Exp | yes | B | Run2 | 9 | 5 | 1.52 | 0.85 | 0.667 | 0.111 | 1 | 1 | 1 | 1 |
| November | 2013 | 1Exp | yes | B | Run3 | 282 | 8 | 0.29 | 0.14 | 0.014 | 0.947 | 1 | 1 | 1 | 1 |
| November | 2013 | 2Con | yes | B | Run1 | 238 | 19 | 1.81 | 0.19 | 0.336 | 0.479 | 5 | 29 | 3 | 16 |
| November | 2013 | 2Con | yes | B | Run2 | 292 | 20 | 1.76 | 0.15 | 0.202 | 0.558 | 6 | 48 | 3 | 16 |
| November | 2013 | 2Con | yes | B | Run3 | 117 | 11 | 1.42 | 0.26 | 0.402 | 0.487 | 4 | 10 | 2 | 2 |
| December | 2013 | 1Exp | no | A | Run1 | 103 | 7 | 1.19 | 0.35 | 0.709 | 0.252 | 3 | 9 | 2 | 2 |
| December | 2013 | 1Exp | no | A | Run2 | 5 | 2 | 0.50 | 0.74 | 0.200 | 0.800 | 0 | 0 | 0 | 0 |
| December | 2013 | 1Exp | no | A | Run3 | 13 | 2 | 0.27 | 0.58 | 0.000 | 0.923 | 0 | 0 | 0 | 0 |
| December | 2013 | 2Con | no | A | Run1 | 11 | 2 | 0.31 | 0.60 | 0.000 | 0.909 | 0 | 0 | 0 | 0 |
| December | 2013 | 2Con | no | A | Run2 | 118 | 12 | 1.35 | 0.23 | 0.508 | 0.407 | 3 | 8 | 1 | 2 |
| December | 2013 | 2Con | no | A | Run3 | 23 | 2 | 0.18 | 0.55 | 0.043 | 0.957 | 1 | 1 | 1 | 1 |
| January | 2014 | 1Exp | no | B | Run1 | 16 | 3 | 0.46 | 0.43 | 0.125 | 0.875 | 1 | 1 | 1 | 1 |
| January | 2014 | 1Exp | no | B | Run2 | 84 | 5 | 0.39 | 0.24 | 0.036 | 0.917 | 1 | 2 | 1 | 2 |
| January | 2014 | 1Exp | no | B | Run3 | 374 | 5 | 0.29 | 0.23 | 0.032 | 0.941 | 1 | 12 | 1 | 12 |
| January | 2014 | 2Con | no | B | Run1 | 199 | 14 | 1.10 | 0.14 | 0.729 | 0.231 | 6 | 14 | 3 | 5 |
| January | 2014 | 2Con | no | B | Run2 | 66 | 4 | 0.76 | 0.44 | 0.742 | 0.242 | 2 | 3 | 1 | 2 |
| January | 2014 | 2Con | no | B | Run3 | 47 | 6 | 1.10 | 0.36 | 0.723 | 0.213 | 2 | 4 | 1 | 2 |

**Table A4** Site scores from the non-metric multidimensional scaling (NMS) axis 1 and axis 2 and the percent of each habit guild within individual samples from runs within a class site (1Exp) and an unused site (2Con) within Alum Creek, Ohio from February 2013 to January 2014.

| **Month** | **Year** | **SiteTrt** | **SA period** | **Plot** | **Sample#** | **NMS Axis 1 site scores** | **NMS Axis 2 site scores** | **Percent Burrowers** | **Percent Climbers** | **Percent Clingers** | **Percent Skaters** | **Percent Sprawlers** | **Percent Swimmers** |
| --- | --- | --- | --- | --- | --- | --- | --- | --- | --- | --- | --- | --- | --- |
| February | 2013 | 1Exp | no | A | 1sRun1 | 1.683 | 0.376 | 0.170 | 0.000 | 0.080 | 0.000 | 0.750 | 0.000 |
| February | 2013 | 1Exp | no | A | 1sRun2 | -0.593 | 0.248 | 1.000 | 0.000 | 0.000 | 0.000 | 0.000 | 0.000 |
| February | 2013 | 1Exp | no | A | 1sRun3 | 0.327 | 0.351 | 0.700 | 0.000 | 0.000 | 0.000 | 0.300 | 0.000 |
| February | 2013 | 2Con | no | A | 2sRun1 | -0.595 | 0.239 | 1.000 | 0.000 | 0.000 | 0.000 | 0.000 | 0.000 |
| February | 2013 | 2Con | no | A | 2sRun2 | 1.169 | 0.687 | 0.190 | 0.000 | 0.000 | 0.000 | 0.810 | 0.000 |
| February | 2013 | 2Con | no | A | 2sRun3 | 0.98 | -0.7 | 0.390 | 0.000 | 0.330 | 0.000 | 0.290 | 0.000 |
| March | 2013 | 1Exp | no | B | 1sRun1 | -0.602 | 0.233 | 0.990 | 0.000 | 0.010 | 0.000 | 0.000 | 0.000 |
| March | 2013 | 1Exp | no | B | 1sRun2 | -0.575 | 0.238 | 1.000 | 0.000 | 0.000 | 0.000 | 0.000 | 0.000 |
| March | 2013 | 1Exp | no | B | 1sRun3 | -0.427 | 0.271 | 0.940 | 0.000 | 0.000 | 0.000 | 0.060 | 0.000 |
| March | 2013 | 2Con | no | B | 2sRun1 | 0.654 | -0.174 | 0.530 | 0.000 | 0.140 | 0.000 | 0.330 | 0.000 |
| March | 2013 | 2Con | no | B | 2sRun2 | 1.363 | 0.486 | 0.260 | 0.030 | 0.060 | 0.000 | 0.650 | 0.010 |
| March | 2013 | 2Con | no | B | 2sRun3 | 0.928 | 0.193 | 0.400 | 0.000 | 0.050 | 0.000 | 0.550 | 0.000 |
| April | 2013 | 1Exp | no | A | 1sRun1 | -0.575 | 0.238 | 1.000 | 0.000 | 0.000 | 0.000 | 0.000 | 0.000 |
| April | 2013 | 1Exp | no | A | 1sRun2 | 0.009 | 0.29 | 0.780 | 0.000 | 0.000 | 0.000 | 0.190 | 0.030 |
| April | 2013 | 1Exp | no | A | 1sRun3 | -0.594 | 0.243 | 1.000 | 0.000 | 0.000 | 0.000 | 0.000 | 0.000 |
| April | 2013 | 2Con | no | A | 2sRun1 | -0.573 | 0.254 | 0.980 | 0.000 | 0.000 | 0.000 | 0.020 | 0.000 |
| April | 2013 | 2Con | no | A | 2sRun2 | 0.061 | 0.135 | 0.740 | 0.000 | 0.050 | 0.000 | 0.200 | 0.010 |
| April | 2013 | 2Con | no | A | 2sRun3 | -0.053 | -0.213 | 0.730 | 0.000 | 0.150 | 0.000 | 0.110 | 0.010 |
| May | 2013 | 1Exp | yes | B | 1sRun1 | -0.446 | 0.206 | 0.940 | 0.000 | 0.020 | 0.000 | 0.040 | 0.000 |
| May | 2013 | 1Exp | yes | B | 1sRun2 | -0.596 | 0.231 | 0.990 | 0.000 | 0.010 | 0.000 | 0.000 | 0.000 |
| May | 2013 | 1Exp | yes | B | 1sRun3 | -0.448 | 0.203 | 0.940 | 0.000 | 0.020 | 0.000 | 0.030 | 0.010 |
| May | 2013 | 2Con | yes | B | 2sRun1 | -0.213 | -0.298 | 0.760 | 0.000 | 0.180 | 0.000 | 0.040 | 0.020 |
| May | 2013 | 2Con | yes | B | 2sRun2 | -0.315 | 0.05 | 0.870 | 0.000 | 0.070 | 0.000 | 0.050 | 0.010 |
| May | 2013 | 2Con | yes | B | 2sRun3 | -0.126 | 0.18 | 0.820 | 0.000 | 0.030 | 0.000 | 0.140 | 0.000 |
| June | 2013 | 1Exp | yes | A | 1sRun1 | -0.34 | -0.194 | 0.830 | 0.000 | 0.170 | 0.000 | 0.000 | 0.000 |
| continued |  |  |  |  |  |  |  |  |  |  |  |  |  |
| **Month** | **Year** | **SiteTrt** | **SA period** | **Plot** | **Sample#** | **NMS Axis 1 site scores** | **NMS Axis 2 site scores** | **Percent Burrowers** | **Percent Climbers** | **Percent Clingers** | **Percent Skaters** | **Percent Sprawlers** | **Percent Swimmers** |
| June | 2013 | 1Exp | yes | A | 1sRun2 | -0.293 | 0.006 | 0.860 | 0.000 | 0.090 | 0.000 | 0.060 | 0.000 |
| June | 2013 | 1Exp | yes | A | 1sRun3 | -0.48 | 0.152 | 0.950 | 0.000 | 0.050 | 0.000 | 0.000 | 0.000 |
| June | 2013 | 2Con | yes | A | 2sRun1 | -0.593 | 0.208 | 0.980 | 0.000 | 0.020 | 0.000 | 0.000 | 0.000 |
| June | 2013 | 2Con | yes | A | 2sRun2 | -0.599 | 0.242 | 0.990 | 0.000 | 0.000 | 0.000 | 0.000 | 0.000 |
| June | 2013 | 2Con | yes | A | 2sRun3 | -0.413 | 0.035 | 0.910 | 0.000 | 0.080 | 0.000 | 0.010 | 0.000 |
| July | 2013 | 1Exp | no | B | 1sRun1 | -0.426 | -0.014 | 0.900 | 0.000 | 0.100 | 0.000 | 0.000 | 0.000 |
| July | 2013 | 1Exp | no | B | 1sRun2 | -0.079 | -0.727 | 0.670 | 0.000 | 0.330 | 0.000 | 0.000 | 0.000 |
| July | 2013 | 1Exp | no | B | 1sRun3 | -0.586 | 0.211 | 0.990 | 0.000 | 0.010 | 0.000 | 0.000 | 0.000 |
| July | 2013 | 2Con | no | B | 2sRun1 | -0.046 | -0.712 | 0.660 | 0.000 | 0.270 | 0.000 | 0.010 | 0.060 |
| July | 2013 | 2Con | no | B | 2sRun2 | -0.191 | -0.229 | 0.780 | 0.000 | 0.160 | 0.000 | 0.050 | 0.010 |
| July | 2013 | 2Con | no | B | 2sRun3 | -0.249 | -0.265 | 0.780 | 0.000 | 0.180 | 0.000 | 0.030 | 0.010 |
| August | 2013 | 1Exp | no | A | 1sRun1 | -0.159 | -0.325 | 0.750 | 0.000 | 0.200 | 0.000 | 0.050 | 0.000 |
| August | 2013 | 1Exp | no | A | 1sRun2 | -0.353 | -0.148 | 0.840 | 0.010 | 0.140 | 0.000 | 0.000 | 0.000 |
| August | 2013 | 1Exp | no | A | 1sRun3 | -0.575 | 0.238 | 1.000 | 0.000 | 0.000 | 0.000 | 0.000 | 0.000 |
| August | 2013 | 2Con | no | A | 2sRun1 | -0.426 | -0.014 | 0.900 | 0.000 | 0.100 | 0.000 | 0.000 | 0.000 |
| August | 2013 | 2Con | no | A | 2sRun2 | 0.319 | -1.082 | 0.460 | 0.030 | 0.490 | 0.000 | 0.030 | 0.000 |
| August | 2013 | 2Con | no | A | 2sRun3 | -0.338 | -0.007 | 0.870 | 0.000 | 0.090 | 0.000 | 0.040 | 0.000 |
| September | 2013 | 1Exp | no | B | 1sRun1 | -0.54 | 0.181 | 0.970 | 0.000 | 0.030 | 0.000 | 0.000 | 0.000 |
| September | 2013 | 1Exp | no | B | 1sRun2 | -0.594 | 0.252 | 1.000 | 0.000 | 0.000 | 0.000 | 0.000 | 0.000 |
| September | 2013 | 1Exp | no | B | 1sRun3 | -0.592 | 0.246 | 0.990 | 0.000 | 0.000 | 0.000 | 0.010 | 0.000 |
| September | 2013 | 2Con | no | B | 2sRun1 | 0.099 | -0.937 | 0.580 | 0.020 | 0.270 | 0.000 | 0.010 | 0.120 |
| September | 2013 | 2Con | no | B | 2sRun2 | 0.133 | -0.931 | 0.540 | 0.000 | 0.350 | 0.000 | 0.010 | 0.100 |
| September | 2013 | 2Con | no | B | 2sRun3 | 0.067 | -0.891 | 0.590 | 0.000 | 0.270 | 0.010 | 0.010 | 0.120 |
| October | 2013 | 1Exp | yes | A | 1sRun1 | -0.225 | -0.022 | 0.830 | 0.000 | 0.090 | 0.000 | 0.060 | 0.010 |
| October | 2013 | 1Exp | yes | A | 1sRun2 | 0.152 | -0.218 | 0.700 | 0.000 | 0.130 | 0.000 | 0.140 | 0.030 |
| October | 2013 | 1Exp | yes | A | 1sRun3 | 0.115 | -0.565 | 0.630 | 0.020 | 0.210 | 0.020 | 0.090 | 0.040 |
| October | 2013 | 2Con | yes | A | 2sRun1 | 0.22 | -0.087 | 0.690 | 0.000 | 0.120 | 0.000 | 0.190 | 0.000 |
| October | 2013 | 2Con | yes | A | 2sRun2 | 0.583 | -0.885 | 0.430 | 0.030 | 0.340 | 0.000 | 0.140 | 0.060 |
| continued |  |  |  |  |  |  |  |  |  |  |  |  |  |
| **Month** | **Year** | **SiteTrt** | **SA period** | **Plot** | **Sample#** | **NMS Axis 1 site scores** | **NMS Axis 2 site scores** | **Percent Burrowers** | **Percent Climbers** | **Percent Clingers** | **Percent Skaters** | **Percent Sprawlers** | **Percent Swimmers** |
| October | 2013 | 2Con | yes | A | 2sRun3 | 0.59 | -0.74 | 0.450 | 0.020 | 0.310 | 0.000 | 0.180 | 0.040 |
| November | 2013 | 1Exp | yes | B | 1sRun1 | -0.426 | -0.014 | 0.900 | 0.000 | 0.100 | 0.000 | 0.000 | 0.000 |
| November | 2013 | 1Exp | yes | B | 1sRun2 | 1.803 | 0.211 | 0.140 | 0.000 | 0.140 | 0.000 | 0.710 | 0.000 |
| November | 2013 | 1Exp | yes | B | 1sRun3 | -0.556 | 0.261 | 0.980 | 0.000 | 0.000 | 0.000 | 0.020 | 0.000 |
| November | 2013 | 2Con | yes | B | 2sRun1 | 0.636 | -0.186 | 0.550 | 0.010 | 0.140 | 0.000 | 0.300 | 0.010 |
| November | 2013 | 2Con | yes | B | 2sRun2 | 0.011 | -0.394 | 0.680 | 0.020 | 0.190 | 0.000 | 0.090 | 0.020 |
| November | 2013 | 2Con | yes | B | 2sRun3 | 0.663 | -0.006 | 0.510 | 0.020 | 0.090 | 0.000 | 0.370 | 0.010 |
| December | 2013 | 1Exp | no | A | 1sRun1 | 1.568 | 0.453 | 0.260 | 0.000 | 0.090 | 0.000 | 0.650 | 0.000 |
| December | 2013 | 1Exp | no | A | 1sRun2 | -0.053 | 0.329 | 0.800 | 0.000 | 0.000 | 0.000 | 0.200 | 0.000 |
| December | 2013 | 1Exp | no | A | 1sRun3 | -0.575 | 0.238 | 1.000 | 0.000 | 0.000 | 0.000 | 0.000 | 0.000 |
| December | 2013 | 2Con | no | A | 2sRun1 | -0.575 | 0.238 | 1.000 | 0.000 | 0.000 | 0.000 | 0.000 | 0.000 |
| December | 2013 | 2Con | no | A | 2sRun2 | 0.84 | 0.114 | 0.440 | 0.010 | 0.070 | 0.000 | 0.470 | 0.010 |
| December | 2013 | 2Con | no | A | 2sRun3 | -0.514 | 0.155 | 0.960 | 0.000 | 0.040 | 0.000 | 0.000 | 0.000 |
| January | 2014 | 1Exp | no | B | 1sRun1 | -0.319 | 0.088 | 0.880 | 0.000 | 0.060 | 0.000 | 0.060 | 0.000 |
| January | 2014 | 1Exp | no | B | 1sRun2 | -0.468 | 0.203 | 0.950 | 0.000 | 0.020 | 0.000 | 0.020 | 0.000 |
| January | 2014 | 1Exp | no | B | 1sRun3 | -0.517 | 0.183 | 0.960 | 0.000 | 0.030 | 0.000 | 0.010 | 0.000 |
| January | 2014 | 2Con | no | B | 2sRun1 | 1.399 | 0.487 | 0.230 | 0.000 | 0.070 | 0.000 | 0.690 | 0.010 |
| January | 2014 | 2Con | no | B | 2sRun2 | 1.226 | 0.498 | 0.240 | 0.000 | 0.050 | 0.000 | 0.710 | 0.000 |
| January | 2014 | 2Con | no | B | 2sRun3 | 1.229 | 0.392 | 0.230 | 0.000 | 0.090 | 0.000 | 0.680 | 0.000 |

**Table A5** Univariate response variables from samples collected from pools within a class site (1Exp) and an unused site (2Con) within Alum Creek, Ohio from February 2013 to December 2013.

| **Month** | **Year** | **SiteTrt** | **SA period** | **Plot** | **Sample#** | **Abundance** | **Taxa Richness** | **Shannon Diversity Index** | **Evenness** | **Percent EPT** | **Percent Chironomidae** | **Clinger Taxa Richness** | **Clinger Abundance** | **Trichoptera Taxa Richness** | **Trichoptera Abundance** |
| --- | --- | --- | --- | --- | --- | --- | --- | --- | --- | --- | --- | --- | --- | --- | --- |
| February | 2013 | 1Exp | no | A | Pool1 | 107 | 4 | 0.347 | 0.291 | 0.047 | 0.925 | 0 | 0 | 0 | 0 |
| February | 2013 | 1Exp | no | A | Pool2 | 40 | 3 | 0.490 | 0.451 | 0.150 | 0.850 | 1 | 5 | 0 | 0 |
| February | 2013 | 1Exp | no | A | Pool3 | 53 | 11 | 1.649 | 0.314 | 0.434 | 0.472 | 3 | 7 | 0 | 0 |
| February | 2013 | 2Con | no | A | Pool1 | 26 | 6 | 0.900 | 0.276 | 0.115 | 0.769 | 0 | 0 | 0 | 0 |
| February | 2013 | 2Con | no | A | Pool2 | 45 | 12 | 2.056 | 0.484 | 0.378 | 0.000 | 2 | 7 | 0 | 0 |
| February | 2013 | 2Con | no | A | Pool3 | 38 | 9 | 1.563 | 0.330 | 0.053 | 0.553 | 0 | 0 | 0 | 0 |
| March | 2013 | 1Exp | no | B | Pool1 | 105 | 6 | 0.689 | 0.244 | 0.086 | 0.819 | 1 | 1 | 0 | 0 |
| March | 2013 | 1Exp | no | B | Pool2 | 114 | 4 | 0.343 | 0.294 | 0.053 | 0.921 | 1 | 1 | 0 | 0 |
| March | 2013 | 1Exp | no | B | Pool3 | 95 | 14 | 1.707 | 0.240 | 0.158 | 0.505 | 3 | 14 | 2 | 22 |
| March | 2013 | 2Con | no | B | Pool1 | 151 | 8 | 1.056 | 0.286 | 0.298 | 0.589 | 3 | 3 | 0 | 0 |
| March | 2013 | 2Con | no | B | Pool2 | 31 | 2 | 0.143 | 0.533 | 0.000 | 0.968 | 0 | 0 | 0 | 0 |
| March | 2013 | 2Con | no | B | Pool3 | 55 | 5 | 0.838 | 0.333 | 0.127 | 0.764 | 1 | 3 | 0 | 0 |
| April | 2013 | 1Exp | no | A | Pool1 | 268 | 4 | 0.207 | 0.272 | 0.030 | 0.959 | 0 | 0 | 0 | 0 |
| April | 2013 | 1Exp | no | A | Pool2 | 193 | 8 | 0.785 | 0.201 | 0.047 | 0.772 | 3 | 5 | 2 | 2 |
| April | 2013 | 1Exp | no | A | Pool3 | 1243 | 9 | 0.282 | 0.125 | 0.049 | 0.944 | 4 | 14 | 2 | 2 |
| April | 2013 | 2Con | no | A | Pool1 | 219 | 6 | 0.882 | 0.311 | 0.228 | 0.699 | 2 | 5 | 0 | 0 |
| April | 2013 | 2Con | no | A | Pool2 | 483 | 11 | 1.019 | 0.181 | 0.232 | 0.675 | 4 | 16 | 2 | 2 |
| April | 2013 | 2Con | no | A | Pool3 | 208 | 7 | 0.785 | 0.229 | 0.197 | 0.774 | 3 | 9 | 0 | 0 |
| May | 2013 | 1Exp | yes | B | Pool1 | 242 | 6 | 0.376 | 0.197 | 0.008 | 0.917 | 1 | 1 | 0 | 0 |
| May | 2013 | 1Exp | yes | B | Pool2 | 219 | 8 | 0.577 | 0.170 | 0.114 | 0.849 | 2 | 2 | 0 | 0 |
| May | 2013 | 1Exp | yes | B | Pool3 | 539 | 7 | 0.140 | 0.149 | 0.011 | 0.978 | 0 | 0 | 0 | 0 |
| May | 2013 | 2Con | yes | B | Pool1 | 199 | 3 | 0.323 | 0.399 | 0.085 | 0.910 | 0 | 0 | 0 | 0 |
| May | 2013 | 2Con | yes | B | Pool2 | 179 | 5 | 0.526 | 0.266 | 0.101 | 0.860 | 1 | 3 | 0 | 0 |
|  |  |  |  |  |  |  |  |  |  |  |  |  |  |  |  |
|  |  |  |  |  |  |  |  |  |  |  |  |  |  |  |  |
| continued |  |  |  |  |  |  |  |  |  |  |  |  |  |  |  |
| **Month** | **Year** | **SiteTrt** | **SA period** | **Plot** | **Sample#** | **Abundance** | **Taxa Richness** | **Shannon Diversity Index** | **Evenness** | **Percent EPT** | **Percent Chironomidae** | **Clinger Taxa Richness** | **Clinger Abundance** | **Trichoptera Taxa Richness** | **Trichoptera Abundance** |
| May | 2013 | 2Con | yes | B | Pool3 | 112 | 2 | 0.051 | 0.509 | 0.009 | 0.991 | 0 | 0 | 0 | 0 |
| June | 2013 | 1Exp | yes | A | Pool1 | 310 | 7 | 0.130 | 0.149 | 0.006 | 0.981 | 1 | 1 | 0 | 0 |
| June | 2013 | 1Exp | yes | A | Pool2 | 414 | 6 | 0.181 | 0.178 | 0.014 | 0.969 | 2 | 5 | 0 | 0 |
| June | 2013 | 1Exp | yes | A | Pool3 | 173 | 5 | 0.243 | 0.220 | 0.023 | 0.954 | 1 | 2 | 0 | 0 |
| June | 2013 | 2Con | yes | A | Pool1 | 173 | 6 | 0.256 | 0.183 | 0.023 | 0.954 | 1 | 1 | 0 | 0 |
| June | 2013 | 2Con | yes | A | Pool2 | 147 | 3 | 0.113 | 0.347 | 0.000 | 0.980 | 1 | 2 | 0 | 0 |
| June | 2013 | 2Con | yes | A | Pool3 | 433 | 8 | 0.270 | 0.137 | 0.014 | 0.954 | 1 | 2 | 0 | 0 |
| July | 2013 | 1Exp | no | B | Pool1 | 175 | 5 | 0.141 | 0.209 | 0.006 | 0.977 | 2 | 2 | 2 | 2 |
| July | 2013 | 1Exp | no | B | Pool2 | 186 | 5 | 0.134 | 0.209 | 0.011 | 0.978 | 0 | 0 | 2 | 2 |
| July | 2013 | 1Exp | no | B | Pool3 | 140 | 2 | 0.042 | 0.507 | 0.000 | 0.993 | 0 | 0 | 0 | 0 |
| July | 2013 | 2Con | no | B | Pool1 | 73 | 2 | 0.126 | 0.528 | 0.000 | 0.973 | 0 | 0 | 0 | 0 |
| July | 2013 | 2Con | no | B | Pool2 | 106 | 8 | 0.411 | 0.146 | 0.009 | 0.925 | 2 | 2 | 0 | 0 |
| July | 2013 | 2Con | no | B | Pool3 | 24 | 6 | 1.310 | 0.480 | 0.042 | 0.500 | 0 | 0 | 0 | 0 |
| August | 2013 | 1Exp | no | A | Pool1 | 17 | 5 | 0.998 | 0.383 | 0.765 | 0.000 | 2 | 3 | 0 | 0 |
| August | 2013 | 1Exp | no | A | Pool2 | 103 | 9 | 0.550 | 0.221 | 0.029 | 0.893 | 0 | 0 | 0 | 0 |
| August | 2013 | 1Exp | no | A | Pool3 | 107 | 10 | 1.163 | 0.196 | 0.140 | 0.701 | 2 | 6 | 2 | 2 |
| August | 2013 | 2Con | no | A | Pool1 | 167 | 12 | 0.702 | 0.113 | 0.078 | 0.856 | 3 | 7 | 0 | 0 |
| August | 2013 | 2Con | no | A | Pool2 | 389 | 8 | 0.575 | 0.178 | 0.167 | 0.823 | 2 | 4 | 2 | 4 |
| August | 2013 | 2Con | no | A | Pool3 | 453 | 3 | 0.162 | 0.356 | 0.026 | 0.967 | 0 | 0 | 0 | 0 |
| September | 2013 | 1Exp | no | B | Pool1 | 2098 | 20 | 0.549 | 0.062 | 0.033 | 0.894 | 3 | 12 | 3 | 26 |
| September | 2013 | 1Exp | no | B | Pool2 | 405 | 11 | 0.636 | 0.120 | 0.037 | 0.867 | 2 | 6 | 0 | 0 |
| September | 2013 | 1Exp | no | B | Pool3 | 228 | 8 | 0.829 | 0.193 | 0.044 | 0.798 | 1 | 1 | 0 | 0 |
| September | 2013 | 2Con | no | B | Pool1 | 51 | 4 | 0.922 | 0.542 | 0.333 | 0.588 | 0 | 0 | 0 | 0 |
| September | 2013 | 2Con | no | B | Pool2 | 273 | 11 | 0.783 | 0.135 | 0.132 | 0.817 | 2 | 18 | 0 | 0 |
| September | 2013 | 2Con | no | B | Pool3 | 201 | 9 | 0.553 | 0.141 | 0.075 | 0.886 | 1 | 1 | 0 | 0 |
| October | 2013 | 1Exp | yes | A | Pool1 | 373 | 13 | 1.077 | 0.145 | 0.214 | 0.716 | 3 | 48 | 2 | 2 |
| October | 2013 | 1Exp | yes | A | Pool2 | 119 | 10 | 1.392 | 0.266 | 0.151 | 0.580 | 2 | 18 | 0 | 0 |
| continued |  |  |  |  |  |  |  |  |  |  |  |  |  |  |  |
| **Month** | **Year** | **SiteTrt** | **SA period** | **Plot** | **Sample#** | **Abundance** | **Taxa Richness** | **Shannon Diversity Index** | **Evenness** | **Percent EPT** | **Percent Chironomidae** | **Clinger Taxa Richness** | **Clinger Abundance** | **Trichoptera Taxa Richness** | **Trichoptera Abundance** |
| October | 2013 | 1Exp | yes | A | Pool3 | 126 | 12 | 1.226 | 0.173 | 0.190 | 0.675 | 3 | 6 | 2 | 2 |
| October | 2013 | 2Con | yes | A | Pool1 | 400 | 16 | 1.462 | 0.193 | 0.423 | 0.435 | 3 | 39 | 3 | 4 |
| October | 2013 | 2Con | yes | A | Pool2 | 584 | 9 | 0.877 | 0.214 | 0.670 | 0.279 | 2 | 11 | 0 | 0 |
| October | 2013 | 2Con | yes | A | Pool3 | 400 | 8 | 1.027 | 0.287 | 0.528 | 0.408 | 2 | 7 | 0 | 0 |
| November | 2013 | 1Exp | yes | B | Pool1 | 236 | 8 | 1.133 | 0.290 | 0.051 | 0.589 | 1 | 8 | 2 | 4 |
| November | 2013 | 1Exp | yes | B | Pool2 | 151 | 10 | 0.667 | 0.134 | 0.066 | 0.861 | 3 | 5 | 3 | 6 |
| November | 2013 | 1Exp | yes | B | Pool3 | 261 | 11 | 1.059 | 0.168 | 0.096 | 0.720 | 3 | 12 | 0 | 0 |
| November | 2013 | 2Con | yes | B | Pool1 | 127 | 14 | 1.581 | 0.241 | 0.165 | 0.378 | 1 | 4 | 0 | 0 |
| November | 2013 | 2Con | yes | B | Pool2 | 16 | 4 | 0.918 | 0.485 | 0.250 | 0.688 | 1 | 1 | 0 | 0 |
| November | 2013 | 2Con | yes | B | Pool3 | 13 | 3 | 0.790 | 0.619 | 0.308 | 0.692 | 0 | 0 | 0 | 0 |
| December | 2013 | 1Exp | no | A | Pool1 | 19 | 6 | 1.484 | 0.573 | 0.421 | 0.474 | 2 | 4 | 0 | 0 |
| December | 2013 | 1Exp | no | A | Pool2 | 149 | 11 | 1.464 | 0.253 | 0.302 | 0.564 | 1 | 19 | 0 | 0 |
| December | 2013 | 1Exp | no | A | Pool3 | 215 | 10 | 0.540 | 0.125 | 0.084 | 0.893 | 4 | 10 | 2 | 2 |
| December | 2013 | 2Con | no | A | Pool1 | 321 | 17 | 1.769 | 0.243 | 0.259 | 0.374 | 4 | 37 | 0 | 0 |
| December | 2013 | 2Con | no | A | Pool2 | 186 | 7 | 0.575 | 0.197 | 0.134 | 0.844 | 2 | 4 | 0 | 0 |
| December | 2013 | 2Con | no | A | Pool3 | 68 | 3 | 0.407 | 0.422 | 0.103 | 0.882 | 0 | 0 | 0 | 0 |

**Table A6** Site scores from the non-metric multidimensional scaling (NMS) axis 1 and the percent of each habit guild within individual samples from pools within a class site (1Exp) and an unused site (2Con) within Alum Creek, Ohio from February 2013 to December 2013.

| **Month** | **Year** | **SiteTrt** | **SA period** | **Plot** | **Sample#** | **NMS Axis 1 site scores** | **Percent Burrowers** | **Percent Climbers** | **Percent Clingers** | **Percent Skaters** | **Percent Sprawlers** | **Percent Swimmers** |
| --- | --- | --- | --- | --- | --- | --- | --- | --- | --- | --- | --- | --- |
| February | 2013 | 1Exp | no | A | Pool1 | 0.415 | 0.925 | 0.000 | 0.000 | 0.000 | 0.075 | 0.000 |
| February | 2013 | 1Exp | no | A | Pool2 | 0.234 | 0.850 | 0.000 | 0.125 | 0.000 | 0.025 | 0.000 |
| February | 2013 | 1Exp | no | A | Pool3 | -1.396 | 0.509 | 0.019 | 0.132 | 0.000 | 0.283 | 0.057 |
| February | 2013 | 2Con | no | A | Pool1 | -0.115 | 0.800 | 0.000 | 0.000 | 0.000 | 0.160 | 0.040 |
| February | 2013 | 2Con | no | A | Pool2 | -3.646 | 0.036 | 0.071 | 0.250 | 0.000 | 0.571 | 0.071 |
| February | 2013 | 2Con | no | A | Pool3 | 0.024 | 0.838 | 0.000 | 0.000 | 0.000 | 0.162 | 0.000 |
| March | 2013 | 1Exp | no | B | Pool1 | 0.046 | 0.837 | 0.000 | 0.010 | 0.000 | 0.154 | 0.000 |
| March | 2013 | 1Exp | no | B | Pool2 | 0.430 | 0.921 | 0.000 | 0.009 | 0.000 | 0.053 | 0.018 |
| March | 2013 | 1Exp | no | B | Pool3 | -0.858 | 0.650 | 0.000 | 0.175 | 0.000 | 0.175 | 0.000 |
| March | 2013 | 2Con | no | B | Pool1 | 1.221 | 0.593 | 0.000 | 0.020 | 0.000 | 0.387 | 0.000 |
| March | 2013 | 2Con | no | B | Pool2 | 0.598 | 0.968 | 0.000 | 0.000 | 0.000 | 0.032 | 0.000 |
| March | 2013 | 2Con | no | B | Pool3 | -0.283 | 0.764 | 0.000 | 0.055 | 0.000 | 0.182 | 0.000 |
| April | 2013 | 1Exp | no | A | Pool1 | 0.567 | 0.959 | 0.000 | 0.000 | 0.000 | 0.037 | 0.004 |
| April | 2013 | 1Exp | no | A | Pool2 | -0.160 | 0.792 | 0.000 | 0.026 | 0.000 | 0.182 | 0.000 |
| April | 2013 | 1Exp | no | A | Pool3 | 0.512 | 0.944 | 0.001 | 0.011 | 0.000 | 0.043 | 0.002 |
| April | 2013 | 2Con | no | A | Pool1 | -0.674 | 0.699 | 0.005 | 0.023 | 0.000 | 0.274 | 0.000 |
| April | 2013 | 2Con | no | A | Pool2 | -0.723 | 0.685 | 0.006 | 0.033 | 0.000 | 0.275 | 0.000 |
| April | 2013 | 2Con | no | A | Pool3 | -0.223 | 0.774 | 0.000 | 0.043 | 0.000 | 0.168 | 0.014 |
| May | 2013 | 1Exp | yes | B | Pool1 | 0.607 | 0.969 | 0.000 | 0.004 | 0.000 | 0.026 | 0.000 |
| May | 2013 | 1Exp | yes | B | Pool2 | 0.107 | 0.849 | 0.009 | 0.009 | 0.000 | 0.132 | 0.000 |
| May | 2013 | 1Exp | yes | B | Pool3 | 0.650 | 0.983 | 0.000 | 0.000 | 0.000 | 0.017 | 0.000 |
| May | 2013 | 2Con | yes | B | Pool1 | 0.350 | 0.910 | 0.000 | 0.000 | 0.000 | 0.090 | 0.000 |
| May | 2013 | 2Con | yes | B | Pool2 | 0.147 | 0.860 | 0.006 | 0.017 | 0.000 | 0.117 | 0.000 |
| May | 2013 | 2Con | yes | B | Pool3 | 0.685 | 0.991 | 0.000 | 0.000 | 0.000 | 0.009 | 0.000 |
| June | 2013 | 1Exp | yes | A | Pool1 | 0.690 | 0.990 | 0.000 | 0.003 | 0.000 | 0.003 | 0.003 |
| June | 2013 | 1Exp | yes | A | Pool2 | 0.624 | 0.971 | 0.000 | 0.012 | 0.002 | 0.015 | 0.000 |
| Continued |  |  |  |  |  |  |  |  |  |  |  |  |
| **Month** | **Year** | **SiteTrt** | **SA period** | **Plot** | **Sample#** | **NMS Axis 1 site scores** | **Percent Burrowers** | **Percent Climbers** | **Percent Clingers** | **Percent Skaters** | **Percent Sprawlers** | **Percent Swimmers** |
| June | 2013 | 1Exp | yes | A | Pool3 | 0.588 | 0.959 | 0.000 | 0.012 | 0.006 | 0.023 | 0.000 |
| June | 2013 | 2Con | yes | A | Pool1 | 0.641 | 0.965 | 0.006 | 0.006 | 0.000 | 0.006 | 0.018 |
| June | 2013 | 2Con | yes | A | Pool2 | 0.667 | 0.980 | 0.000 | 0.014 | 0.007 | 0.000 | 0.000 |
| June | 2013 | 2Con | yes | A | Pool3 | 0.663 | 0.979 | 0.000 | 0.005 | 0.002 | 0.007 | 0.007 |
| July | 2013 | 1Exp | no | B | Pool1 | 0.665 | 0.983 | 0.000 | 0.011 | 0.000 | 0.006 | 0.000 |
| July | 2013 | 1Exp | no | B | Pool2 | 0.687 | 0.989 | 0.005 | 0.000 | 0.000 | 0.005 | 0.000 |
| July | 2013 | 1Exp | no | B | Pool3 | 0.687 | 0.993 | 0.000 | 0.000 | 0.000 | 0.007 | 0.000 |
| July | 2013 | 2Con | no | B | Pool1 | 0.674 | 0.973 | 0.000 | 0.000 | 0.027 | 0.000 | 0.000 |
| July | 2013 | 2Con | no | B | Pool2 | 0.533 | 0.942 | 0.000 | 0.019 | 0.010 | 0.029 | 0.000 |
| July | 2013 | 2Con | no | B | Pool3 | -0.545 | 0.600 | 0.000 | 0.000 | 0.350 | 0.050 | 0.000 |
| August | 2013 | 1Exp | no | A | Pool1 | -3.871 | 0.000 | 0.000 | 0.176 | 0.059 | 0.706 | 0.059 |
| August | 2013 | 1Exp | no | A | Pool2 | 0.527 | 0.920 | 0.010 | 0.000 | 0.030 | 0.020 | 0.020 |
| August | 2013 | 1Exp | no | A | Pool3 | -0.181 | 0.773 | 0.010 | 0.062 | 0.010 | 0.113 | 0.031 |
| August | 2013 | 2Con | no | A | Pool1 | 0.224 | 0.873 | 0.006 | 0.042 | 0.000 | 0.072 | 0.006 |
| August | 2013 | 2Con | no | A | Pool2 | 0.006 | 0.827 | 0.005 | 0.010 | 0.000 | 0.155 | 0.003 |
| August | 2013 | 2Con | no | A | Pool3 | 0.605 | 0.967 | 0.000 | 0.000 | 0.007 | 0.026 | 0.000 |
| September | 2013 | 1Exp | no | B | Pool1 | 0.613 | 0.960 | 0.005 | 0.006 | 0.001 | 0.015 | 0.014 |
| September | 2013 | 1Exp | no | B | Pool2 | 0.342 | 0.903 | 0.000 | 0.015 | 0.000 | 0.077 | 0.005 |
| September | 2013 | 1Exp | no | B | Pool3 | 0.252 | 0.873 | 0.000 | 0.005 | 0.024 | 0.094 | 0.005 |
| September | 2013 | 2Con | no | B | Pool1 | 1.251 | 0.588 | 0.000 | 0.000 | 0.000 | 0.392 | 0.020 |
| September | 2013 | 2Con | no | B | Pool2 | 0.014 | 0.826 | 0.000 | 0.067 | 0.000 | 0.096 | 0.011 |
| September | 2013 | 2Con | no | B | Pool3 | 0.389 | 0.909 | 0.000 | 0.005 | 0.005 | 0.066 | 0.015 |
| October | 2013 | 1Exp | yes | A | Pool1 | -0.352 | 0.750 | 0.003 | 0.130 | 0.000 | 0.109 | 0.008 |
| October | 2013 | 1Exp | yes | A | Pool2 | -0.986 | 0.607 | 0.000 | 0.154 | 0.009 | 0.222 | 0.009 |
| October | 2013 | 1Exp | yes | A | Pool3 | -0.384 | 0.714 | 0.008 | 0.050 | 0.059 | 0.160 | 0.008 |
| October | 2013 | 2Con | yes | A | Pool1 | -1.511 | 0.485 | 0.006 | 0.107 | 0.003 | 0.399 | 0.000 |
| October | 2013 | 2Con | yes | A | Pool2 | 2.264 | 0.291 | 0.000 | 0.019 | 0.000 | 0.690 | 0.000 |
| October | 2013 | 2Con | yes | A | Pool3 | 1.884 | 0.427 | 0.000 | 0.018 | 0.000 | 0.554 | 0.000 |
| Continued |  |  |  |  |  |  |  |  |  |  |  |  |
| **Month** | **Year** | **SiteTrt** | **SA period** | **Plot** | **Sample#** | **NMS Axis 1 site scores** | **Percent Burrowers** | **Percent Climbers** | **Percent Clingers** | **Percent Skaters** | **Percent Sprawlers** | **Percent Swimmers** |
| November | 2013 | 1Exp | yes | B | Pool1 | -0.948 | 0.610 | 0.018 | 0.035 | 0.000 | 0.338 | 0.000 |
| November | 2013 | 1Exp | yes | B | Pool2 | 0.453 | 0.922 | 0.000 | 0.035 | 0.000 | 0.035 | 0.007 |
| November | 2013 | 1Exp | yes | B | Pool3 | -0.397 | 0.745 | 0.000 | 0.047 | 0.000 | 0.200 | 0.008 |
| November | 2013 | 2Con | yes | B | Pool1 | -0.107 | 0.772 | 0.031 | 0.031 | 0.000 | 0.071 | 0.094 |
| November | 2013 | 2Con | yes | B | Pool2 | -0.701 | 0.688 | 0.000 | 0.063 | 0.000 | 0.250 | 0.000 |
| November | 2013 | 2Con | yes | B | Pool3 | -0.708 | 0.692 | 0.000 | 0.000 | 0.000 | 0.231 | 0.077 |
| December | 2013 | 1Exp | no | A | Pool1 | -1.568 | 0.474 | 0.000 | 0.211 | 0.000 | 0.316 | 0.000 |
| December | 2013 | 1Exp | no | A | Pool2 | -0.993 | 0.601 | 0.000 | 0.133 | 0.000 | 0.231 | 0.035 |
| December | 2013 | 1Exp | no | A | Pool3 | 0.371 | 0.893 | 0.009 | 0.047 | 0.000 | 0.037 | 0.014 |
| December | 2013 | 2Con | no | A | Pool1 | -1.897 | 0.378 | 0.006 | 0.116 | 0.000 | 0.450 | 0.050 |
| December | 2013 | 2Con | no | A | Pool2 | 0.118 | 0.853 | 0.000 | 0.022 | 0.000 | 0.125 | 0.000 |
| December | 2013 | 2Con | no | A | Pool3 | 0.202 | 0.882 | 0.000 | 0.000 | 0.000 | 0.118 | 0.000 |
